# The Blattodea *s.s.* (Insecta, Dictyoptera) of the Guiana Shield

**DOI:** 10.3897/zookeys.475.7877

**Published:** 2015-01-22

**Authors:** Dominic A. Evangelista, Kimberly Chan, Kayla L. Kaplan, Megan M. Wilson, Jessica L. Ware

**Affiliations:** 1 Department of Biology, Rutgers University, 195University Ave, Newark, NJ, 07102, USA

**Keywords:** Cockroach, species richness, *Calhypnorna*, *Xestoblatta*, Guyana

## Abstract

Here we provide a checklist of cockroach species known from areas within the Guiana Shield based on literature records and new field collection. We give records of sixteen species collected in Guyana, eight of which are new records for Guyana and one of which is a new generic record for the entire Guiana Shield. We also provide a description for a geographically disparate species of *Calhypnorna* Stal, and the new species *Xestoblatta berenbaumae*. The complete checklist contains 234 species of Blattodea *s.s.* currently known in the shield. This checklist shows particularly low richness in Guianan Venezuela, Roraima and Amapa Brazil, but this is likely an artifact due to under–sampling. Indeed, based on previously published data and current fieldwork, we believe that most regions of the Guiana Shield are under–sampled for cockroaches. Despite this, French Guiana (151 spp.) and Suriname (136 spp.) rank as the second and sixth most species dense faunas of cockroaches in the neotropics.

## Introduction

The Guiana Shield is known for a high diversity of both plant and animal life (Alexander et al. 2005). Blattodea (Insecta: Dictyoptera), or cockroaches and termites, as well as most other insects, remain under-sampled relative to their biodiversity in the region. Developing more complete lists of fauna improves our ability to infer biogeographical patterns and make predictions about biodiversity loss. Additionally, keeping current records of regional faunas can assist in documenting introduced and invasive species, something particularly relevant to the study of cockroaches (Evangelista et al. 2013; Nickle 1984; Peterson and Cobb 2009).

The cockroach fauna of the entire Guiana Shield has previously been addressed by three works (i.e. Bonfils 1975; Bruijning 1959; Princis 1963). Princis’ catalogue (1963) of global cockroach distributions is an important resource to consult for this fauna. However, there were cases (although very few) where Princis was incomplete in his records (pers. obs.; Pellens and Grandcolas 2008). Bruijning’s (1959) and Bonfils’ (1975) checklists are more manageable than Princis’s global catalog given their focused geographic scope, but they are also an incomplete record of the fauna. Regardless, Bonfils’ (1975), Bruijning’s (1959) and Princis’ (1963) work are all now 40 years or more out of date.

The cockroach fauna of sections of the Guiana Shield have been addressed directly by a few sources (e.g., Bonfils 1987; Bruijning 1959; Hebard 1926; Perez 1988; Rehn 1906; Rocha E Silva Albuquerque and Gurney 1962) as well as peripherally by others (e.g., Evangelista et al. 2014; Hebard 1921b; 1929; Pellens and Grandcolas 2008; Rehn 1928; Velez 2008). A few manuscripts have addressed the Blattodean faunas of French Guiana (Hebard 1926) and Suriname (Bruijning 1959) respectively. The Guianan fauna of relevant parts of Brazil and Venezuela are available from checklists for these respective countries (Bonfils 1987; Pellens and Grandcolas 2008; Perez 1988). However, there is no singular source to be consulted for the Blattodean fauna of Guyana (formerly known as British Guyana).

Lastly, the most current phylogenies of Blattodea all show that termites (Termitoidae) are nested within Blattodea (Djernaes et al. 2012, 2014; Inward et al. 2007; Ware et al. 2008). Given that this has only been recently adopted by systematists, there are few taxonomic treatments considering both termites and cockroaches simultaneously. Since each insect group requires very different morphological and organismal expertise this is understandable. In following, we present the most recent summative list of the non-termite Blattodea fauna of the Guiana Shield as well as for the country of Guyana.

## Methods

### Checklist

The checklist was initially compiled by synthesizing range data from the published literature. Searches for taxonomic records included some combination of the following locality names: British Guiana, Suriname, French Guiana, Guyane, Guiana or Guyana. Five additional sources were consulted (Bonfils 1987; Lopez-Osorio and Miranda-Esquivel 2010; Pellens and Grandcolas 2008; Perez 1988) for the taxa of the following states: Amazonas Venezuela, Bolivar Venezuela, Delta Amacuro Venezuela, Roraima Brazil and Amapa Brazil. The states of Para and Amazonas in Brazil were omitted because the majority of these states do not fall within the borders of the Guiana Shield. The recently published checklist of the cockroaches of Brazil (Pellens and Grandcolas 2008) sufficiently covered the fauna of these states. We treated ranges specified by Princis (1963) as circumtropical, neotropical, or cosmopolitan as a presence for each region, even without a specific record for that region. Additional records were added based on specimens collected by the Ware lab in the field.

The validity of all taxonomic names was verified on the Cockroach Species File (CSF) online database (Beccaloni 2014). All synonymous names were changed to their valid name in the final checklist. All invalid higher taxa were given proper names in accordance with the most current taxonomy (Beccaloni and Eggleton 2011, 2013).

### Specimen collection

We collected cockroaches from the field on four occasions from 2011 to 2013. All specimens were collected in Guyana. Specific collection information (locality and GPS, collection date, collectors and ecological information) is given with each record.

### New records and descriptions

Species that were collected and could be identified are presented here. We report all collection information and some morphological information for each specimen as well as currently known geographic distribution as described on the Cockroach Species File database (Beccaloni 2014). All morphological measurements were done using Infinity software (INFINITY Camera Software 2013). For new species, we provide descriptions of gross morphology and male genitalia. The genitalia were dissected in accordance with the method of Roth (1969), whereby the genitalia are removed from the specimen by making a lateral incision along the subgenital plate, separating the genitalia from the remainder of the body and placing them in a KOH (10% by mass) solution until cleared (approx. 8 hours). Cleared genitalia were kept in a micro-vial with 70% ethanol after examination. We also include some notes on potential evolutionary relationships of some genera by referencing the cytochrome oxidase I (COI) gene tree published by the first and last author (Evangelista et al. 2014).

We imported the checklist data into Mathematica 9.1 (Wolfram Research 2012) to calculate the endemism rates of the faunas of each region. We calculated this as the proportion of species in a given region not present in any other region of the shield. We also calculated faunal similarity rates (inverse of endemism) among each region.

## Results

### Records and descriptions of cockroaches from Guyana

Here we report information on some of the specimens from our field collection. Those species listed here that are new records for Guyana are denoted by a “+” in the checklist (Table 1). Morphological measurements for all specimens are given in Table 2.

**Table 1.** Checklist of species from 8 regions of the Guiana Shield. ? = Record with a non-specific locality, and thus unconfirmed in this region. o = Presence record from published literature. + = new record from this paper. Amaz VEN = Amazonas Venezuela, Bolivar VEN = Bolivar Venezuela, Del Ama VEN = Delta Amacuro Venezuela, Rora BRA = Roraima Brazil, GUY = Guyana, SUR = Suriname, FG = French Guiana, Amapa BRA = Amapa Brazil.

Taxon	*Amaz VEN*	*Bolivar VEN*	*Del Ama VEN*	*Rora BRA*	*GUY*	*SUR*	*FG*	*Amapa BRA*	Source
**Blaberidae**									
**Blaberinae**									
*Blaberus atropos* (Stoll, 1813)	?	?	?		o				(Bruijning 1959; Hebard 1929; Perez 1988; Princis 1963; Princis and Kevan 1955; Walker 1868)
*Blaberus colosseus* (Illiger, 1801)					o		o		(Bonfils 1987; Princis 1963; Hebard 1916)
*Blaberus craniifer* Burmeister, 1838					o		o		(Princis 1963; Gutierrez and Perez-Gelabert 2000 )
*Blaberus discoidalis* Serville, 1838		o							(Princis 1963; Perez 1988; Princis and Kevan 1955; Gutierrez and Perez-Gelabert 2000)
*Blaberus giganteus* (Linnaeus, 1758)					o	o	o	o	(Bonfils 1975; Bruijning 1959; Gutierrez and Perez-Gelabert 2000; Hebard 1926, 1921b; Pellens and Grandcolas 2008; Perez 1988; Princis 1963; Princis and Kevan 1955; Rocha e Silva Albuquerque and Gurney 1962; Walker 1868)
*Blaberus latissimus* (Herbst, 1786)						o			(Princis 1963)
*Blaberus parabolicus* Walker, 1868						o			(Bruijning 1959; Princis 1963)
*Eublaberus distanti* (Kirby, 1903)					o	o	o	o	(Bruijning 1959; Hebard 1926; Pellens and Grandcolas 2008; Princis 1963)
*Eublaberus posticus* (Erichson, 1848)					o	o	o		(Bruijning 1959; Hebard 1926, 1929; Princis 1963; Princis and Kevan 1955; Rehn 1906)
*Eublaberus sulzeri* (Guerin-Meneville, 1857)						o			(Princis 1963)
*Hormetica laevigata* Burmeister, 1838	o								(Perez 1988)
*Hormetica marmorata* (Saussure, 1869)	?	?	?						(Bonfils 1987; Perez 1988; Princis 1963)
*Lucihormetica verrucosa* (Brunner von Wattenwyl, 1865)	?	?	?						(Perez 1988; Hebard 1929; Princis 1963)
*Neorhicnoda maronensis* (Hebard, 1921)					+	o	o		(Bonfils 1975; Bruijning 1959; Grandcolas 1992b; Hebard 1921b, 1926; Princis 1963)
*Paradicta rotunda* Grandcolas, 1992							o		(Grandcolas 1992b)
*Paradicta circumvagans* (Burmeister, 1838)	o	o	o	o	o	o	o	o	(Princis 1963)
*Phoetalia pallida* (Brunner von Wattenwyl, 1865)	o	o	o	o	o	o	o	o	(Baaren et al. 2002; Bruijning 1959; Princis 1963; Princis and Kevan 1955)
*Sibylloblatta pustulata* (Hebard, 1929)	?	?	?						(Bonfils 1987; Perez 1988)
**Epilamprinae**									
*Colapteroblatta bordoni* Bonfils, 1987		o							(Bonfils 1987)
*Colapteroblatta surinama* (Saussure, 1868)					o	o			(Princis 1963; Roth Gutierrez 1998)
*Dryadoblatta mira* Rehn, 1937	o								(Bonfils 1987; Perez 1988; Princis 1963; Rehn 1937a)
*Epilampra abdomennigrum* (De Geer, 1773)					o	o	o		(Bonfils 1975; Bruijning 1959; Hebard 1921b, 1926, 1929; Princis 1963; Princis and Kevan 1955; Rehn 1903; Shelford 1910)
*Epilampra amapae* Rocha e Silva Albuquerque & Gurney, 1962								o	(Pellens and Grandcolas 2008; Rocha e Silva Albuquerque and Gurney 1962)
*Epilampra azteca* Saussure, 1868	o					o	o		(Bonfils 1975 1987; Bruijning 1959; Hebard 1921b, 1926; Perez 1988; Princis 1963; Princis and Kevan 1955)
*Epilampra bromeliacea* Princis, 1965					o				(Princis 1963)
*Epilampra carsevennae* Bonfils, 1975							o		(Bonfils 1975)
*Epilampra colorata* Rocha e Silva Albuquerque & Gurney, 1962								o	(Pellens and Grandcolas 2008; Rocha e Silva Albuquerque and Gurney 1962)
*Epilampra conferta* Walker, 1868								o	(Pellens and Grandcolas 2008; Rocha e Silva Albuquerque and Gurney 1962)
*Epilampra conspersa* Burmeister, 1868						o	o	o	(Bruijning 1959; Hebard 1921b, 1926; Pellens and Grandcolas 2008; Rocha e Silva Albuquerque and Gurney 1962)
*Epilampra crossea* Saussure, 1864						o	o		(Bonfils 1975; Bruijning 1959; Hebard 1921b, 1926; Princis 1963)
*Epilampra egregia* Hebard, 1926						o	o		(Bonfils 1975; Bruijning 1959; Princis 1963)
*Epilampra fusca* Brunner von Wattenwyl, 1865	?	?	?		o	o			(Bruijning 1959; Perez 1988; Princis 1963; Rehn 1906)
*Epilampra grisea* (De Geer, 1773)					o	o	o	o	(Baaren et al. 2002; Bonfils 1975; Bruijning 1959; Hebard 1921b, 1926; Pellens and Grandcolas 2008; Perez 1988; Princis 1963; Rehn 1903, 1906; Rocha e Silva Albuquerque and Gurney 1962; Shelford 1910; Walker 1868)
*Epilampra guianae* Hebard, 1926					o	o	o		(Bruijning 1959; Hebard 1926; Princis 1963)
*Epilampra maculicollis* (Serville, 1838)					o		o		(Hebard 1921b; Rehn 1906)
*Epilampra opaca* Walker, 1868	?				o	o	o	o	(Bonfils 1975; Bruijning 1959; Hebard 1926, 1929; Pellens and Grandcolas 2008; Perez 1988; Rocha e Silva Albuquerque and Gurney 1962; Roth 1970; Walker 1868)
*Epilampra sagitta* Hebard, 1929								o	(Pellens and Grandcolas 2008; Rocha e Silva Albuquerque and Gurney 1962)
*Epilampra sodalis* Walker, 1868	o				+	o	o	o	(Bonfils 1975; Bruijning 1959; Hebard 1926; Pellens and Grandcolas 2008; Perez 1988; Princis 1963)
*Epilampra substrigata* Walker, 1868	o							o	(Pellens and Grandcolas 2008; Perez 1988; Princis 1963; Roth 1970; Rocha e Silva Albuquerque and Gurney 1962; Shelford 1910)
*Epilampra taira* Hebard, 1926						o	o	o	(Bruijning 1959; Hebard 1926; Pellens and Grandcolas 2008; Princis 1963)
*Galiblatta cribrosa* Hebard, 1926						o	o		(Bruijning 1959; Hebard 1926)
*Notolampra punctata* Saussure, 1862						o	o	o	(Hebard 1926; Pellens and Grandcolas 2008; Princis 1963; Rocha e Silva Albuquerque and Gurney 1962)
*Phoraspis pellucens* (Thunberg, 1826)						o			(Princis 1963; Shelford 1910)
**Oxyhaloinae**									
*Nauphoeta cinerea* (Olivier, 1789)	o	o	o	o	o	o	o	o	(Princis 1963)
*Rhyparobia maderae* (Fabricius, 1781)	o	o	o	o	o	o	o	o	(Bonfils 1987; Bruijning 1959; Hebard 1926; Perez 1988; Princis and Kevan 1955)
**Panchlorinae**									
*Achroblatta luteola* (Blanchard, 1843)					o	o	o		(Bruijning 1959; Hebard 1926; Princis 1963)
*Panchlora aurora* Hebard, 1926					o	o	o		(Bonfils 1975; Bruijning 1959; Hebard 1926; Princis 1963)
*Panchlora bidentula* Hebard, 1916	o					o	o		(Bonfils 1975; Bonfils 1987; Bruijning 1959; Hebard 1926; Perez 1988; Princis 1963; Princis and Kevan 1955)
*Panchlora dumicola* Rocha e Silva Albuquerque & Gurney, 1962								o	(Pellens and Grandcolas 2008; Rocha e Silva Albuquerque and Gurney 1962)
*Panchlora exoleta* Burmeister, 1838	?	?	?					o	(Pellens and Grandcolas 2008; Perez 1988; Rocha e Silva Albuquerque and Gurney 1962)
*Panchlora fraterna* Saussure & Zehntner, 1893					o	o			(Bruijning 1959; Hebard 1929; Princis 1963)
*Panchlora hebardi* Princis, 1951					o	o	o		(Bonfils 1975; Princis 1963)
*Panchlora maracaensis* Lopes & Oliveira, 2000				o					(Pellens and Grandcolas 2008)
*Panchlora nivea* (Linnaeus, 1758)	o	o	o	o	o	o	o	o	(Bonfils 1975; Bruijning 1959; Hebard 1921b, 1926, 1929; Pellens and Grandcolas 2008; Perez 1988; Princis 1963; Princis and Kevan 1955; Walker 1868)
*Panchlora peruana* Saussure, 1864					o				(Rehn 1906)
*Panchlora regalis* Hebard, 1926						o	o	o	(Bruijning 1959; Hebard 1926; Pellens and Grandcolas 2008; Princis 1963; Rocha e Silva Albuquerque and Gurney 1962)
*Panchlora thalassina* Saussure & Zehntner, 1893	o	o						o	(Bonfils 1987; Pellens and Grandcolas 2008; Perez 1988)
*Panchlora viridis* (Fabricius, 1775)					o				(Gutierrez and Perez-Gelabert 2000; Rehn 1906)
**Pycnoscelinae**									
*Proscratea complanata* (Perty, 1832)						o	o		(Bonfils 1975; Bruijning 1959; Hebard 1926; Princis 1963)
*Pycnoscelus surinamensis* (Linnaeus, 1758)	o	o	o	o	o	o	o	o	(Bonfils 1987; Bruijning 1959; Hebard 1926; Perez 1988; Princis 1963; Princis and Kevan 1955; Rehn 1906)
**Zetoborinae**									
*Lanxoblatta emarginata* (Burmeister, 1931)					o	o	o		(Bruijning 1959; Hebard 1921b, 1926; Princis 1963)
*Phortioeca nimbata* (Burmeister, 1838)						o	o		(Bruijning 1959; Hebard 1921b, 1926; Princis 1963)
*Schizopilia fissicollis* (Serville, 1838)						o	o		(Bruijning 1959; Hebard 1921b, 1926; Princis 1963; Rehn and Hebard 1927; Shelford 1910)
*Schizopilia neblinensis* Lindemann, 1971	o								(Beccaloni 2007; Bonfils 1987)
*Schizopilia nitor* Grandcolas, 1991							o		(Baaren et al. 2002; Grandcolas 1990)
*Thanatophyllum akinetum* Grandcolas, 1991					+		o		(Grandcolas 1990)
*Tribonium guyanense* Grandcolas, 1993							o		(Grandcolas 1993b)
*Zetoborella gemmicula* Hebard, 1921						o	o		(Bruijning 1959; Hebard 1921b, 1926; Princis 1963)
**Blattidae**									
**Blattinae**									
*Blatta orientalis* Linnaeus, 1758	o	o	o	o	o	o	o	o	(Princis 1963)
*Neostylopyga rhombifolia* (Stoll, 1813)	o	o	o	o	o	o	o	o	(Perez 1988; Princis 1963)
*Pelmatosilpha guianae* Hebard, 1926						o	o	o	(Baaren et al. 2002; Bonfils 1975; Bruijning 1959; Hebard 1926; Pellens and Grandcolas 2008; Rehn 1930)
*Pelmatosilpha lata* Hebard, 1929					o	o			(Bruijning 1959; Hebard 1929; Princis 1963; Rehn 1930)
*Pelmatosilpha macu* Rehn, 1930								o	(Pellens and Grandcolas 2008; Rocha e Silva Albuquerque and Gurney 1962)
*Pelmatosilpha miranha* Rehn, 1930								o	(Pellens and Grandcolas 2008; Rocha e Silva Albuquerque and Gurney 1962)
*Periplaneta americana* (Linnaeus, 1758)	o	o	o	o	o	o	o	o	(Bruijning 1959; Hebard 1926; Princis 1963; Perez 1988; Princis and Kevan 1955)
*Periplaneta australiasiae* (Fabricius, 1775)	o	o	o	o	o	o	o	o	(Bruijning 1959; Hebard 1926; Princis 1963; Perez 1988; Princis and Kevan 1955; Rocha e Silva Albuquerque and Gurney 1962)
*Periplaneta brunnea* Burmeister, 1838	o	o	o	o	o	o	o	o	(Bruijning 1959; Hebard 1921b, 1926; Perez 1988; Princis 1963; Princis and Kevan 1955)
**Polyzosteriinae**									
*Eurycotis blattoides* Hebard, 1926					o	o	o		(Bruijning 1959; Hebard 1926; Princis 1963)
**Corydiidae**									
**Corydiinae**									
*Eulissosoma stygia* Hebard, 1926						o	o		(Hebard 1926)
**Holocompsinae**									
*Holocompsa nitidula* (Fabricius, 1781)	o	o	o	o	o	o	o	o	(Bruijning 1959; Hebard 1921b, 1926; Princis 1963; Princis and Kevan 1955; Rehn 1906)
**Latindiinae**									
*Buboblatta geijskesi* Bruijning, 1959						o			(Bruijning 1959; Princis 1963)
*Latindia dohrniana* Saussure & Zehntner, 1894	?	?	?	?	?	o	o	?	(Bruijning 1959; Hebard 1921b, 1926; Princis 1963)
**Tiviinae**									
*Melestora fusca* Rocha e Silva Albuquerque, 1964	o								(Bonfils 1987; Perez 1988)
*Oulopteryx dascilloides* Hebard, 1921						o	o		(Bruijning 1959; Hebard 1921b, 1926; Princis 1963)
*Sphecophila polybiarum* Shelford, 1907							o		(Bruijning 1959; Hebard 1921b, 1926)
**Ectobiidae**									
**Anaplectinae**									
*Anaplecta analisignata* Rehn, 1916								o	(Pellens and Grandcolas 2008)
*Anaplecta balachowskyi* Bonfils, 1975							o		(Bonfils 1975)
*Anaplecta bivittata* Brunner von Wattenwyl, 1865	o							o	(Bonfils 1987; Pellens and Grandcolas 2008; Perez 1988; Princis 1963; Rocha e Silva Albuquerque and Gurney 1962)
*Anaplecta guianae* Bruijning, 1959						o			(Bruijning 1959)
*Anaplecta hemiscotia* Hebard, 1920								o	(Pellens and Grandcolas 2008; Rocha e Silva Albuquerque and Gurney 1962)
*Anaplecta jari* Rocha e Silva Albuquerque, 1966								o	(Pellens and Grandcolas 2008)
*Anaplecta lateralis* Burmeister, 1838	o								(Bonfils 1987; Perez 1988; Princis 1963)
*Anaplecta maronensis* Hebard, 1921						o	o	o	(Bruijning 1959; Hebard 1921b; Pellens and Grandcolas 2008)
*Anaplecta minutissima* (De Geer, 1773)						o	o		(Bruijning 1959; Hebard 1926)
*Anaplecta parviceps* (Walker, 1868)					o	o	o	o	(Bruijning 1959; Hebard 1926; Pellens and Grandcolas 2008)
*Anaplecta pluto* Hebard, 1926						o	o		(Bonfils 1975; Bruijning 1959; Hebard 1926; Princis 1963)
*Anaplecta poecila* Hebard, 1926	o				o	o	o	o	(Bonfils 1975; Bruijning 1959; Hebard 1926; Pellens and Grandcolas 2008; Perez 1988; Princis 1963; Rocha e Silva Albuquerque and Gurney 1962)
*Anaplecta pulchella* Rehn, 1906					o	o	o	o	(Bruijning 1959; Hebard 1921b; Pellens and Grandcolas 2008; Rehn 1906)
*Anaplecta pygmaea* Bruijning, 1959						o			(Bruijning 1959; Princis 1963)
*Anaplecta subsignata* Hebard, 1926	o					o	o	o	(Bruijning 1959; Hebard 1926; Pellens and Grandcolas 2008; Perez 1988; Princis 1963; Rocha e Silva Albuquerque and Gurney 1962)
*Anaplecta suffusa* Hebard, 1926					o	o	o	o	(Bruijning 1959; Hebard 1926; Pellens and Grandcolas 2008; Perez 1988; Princis 1963; Rocha e Silva Albuquerque and Gurney 1962)
*Maraca fossata* Hebard, 1926	o					o	o		(Bruijning 1959; Hebard 1926; Perez 1988; Princis 1963)
**Attaphilinae**									
*Attaphila aptera* Bolivar, 1905						o			(Bruijning 1959; Princis 1963)
**Blattellinae**									
*Anisopygia decora* Hebard, 1926					+		o		(Bruijning 1959; Hebard 1926)
*Blattella germanica* (Linnaeus, 1767)	o	o	o	o	o	o	o	o	(Bonfils 1987; Perez 1988; Princis 1963; Princis and Kevan 1955)
*Cahita insignis* (Hebard, 1926)							o		(Bruijning 1959; Hebard 1926; Rehn 1937a)
*Chromatonotus coloratus* Rocha e Silva Albuquerque, 1964	o								(Perez 1988)
*Chromatonotus infuscatus* (Bruner, 1906)	o								(Bonfils 1987; Princis 1963; Princis and Kevan 1955)
*Chromatonotus notatus* (Brunner von Wattenwyl, 1893)						o	o		(Bonfils 1975 1987; Bruijning 1959; Hebard 1926; Princis 1963)
*Dasyblatta charpentierae* Bonfils, 1975							o		(Bonfils 1975)
*Dasyblatta maldonadoi* Rocha e Silva Albuquerque, 1964	o								(Bonfils 1987; Perez 1988; Princis 1963)
*Dasyblatta stylata* Bonfils, 1975							o		(Bonfils 1975)
*Dasyblatta thaumasia* Hebard, 1921						o			(Bonfils 1975; Bruijning 1959; Princis 1963)
*Eudromiella bequaerti* Rehn, 1932	o								(Bonfils 1987; Perez 1988; Princis 1963)
*Eudromiella chopardi* Hebard, 1926						o	o		(Bruijning 1959; Hebard 1926; Princis 1963)
*Eudromiella inexpectata* (Rehn, 1906)					o		o		(Bruijning 1959; Hebard 1926, 1929; Princis 1963; Rehn 1906)
*Eudromiella maroni* Hebard, 1926							o		(Bruijning 1959; Hebard 1926)
*Ischnoptera atrata* Hebard, 1916					o				(Princis 1963; Hebard 1916)
*Ischnoptera castanea* Saussure, 1869	o							o	(Bonfils 1987; Pellens and Grandcolas 2008; Perez 1988; Rocha e Silva Albuquerque and Gurney 1962)
*Ischnoptera clavator* Rehn, 1918								o	(Pellens and Grandcolas 2008; Rocha e Silva Albuquerque and Gurney 1962)
*Ischnoptera galibi* Hebard, 1926						o	o		(Bonfils 1975; Bruijning 1959; Hebard 1926)
*Ischnoptera hercules* Rehn, 1928					o	o			(Bruijning 1959; Princis 1963; Rehn 1928)
*Ischnoptera neoclavator* Rocha e Silva Albuquerque, 1964	o								(Bonfils 1987; Perez 1988; Princis 1963)
*Ischnoptera ocularis* Saussure, 1873							o		(Beccaloni 2007)
*Ischnoptera paramacca* Hebard, 1926					o	o	o		(Bonfils 1975; Bruijning 1959; Hebard 1926; Princis 1963)
*Ischnoptera rehni* Hebard, 1926					o	o	o	o	(Bonfils 1975, 1987; Bruijning 1959; Hebard 1926; Pellens and Grandcolas 2008; Princis 1963; Rocha e Silva Albuquerque and Gurney 1962)
*Ischnoptera rufa* (De Geer, 1773)					o	o			(Bruijning 1959; Gutierrez and Perez-Gelabert 2000; Princis and Kevan 1955; Rehn 1903)
*Ischnoptera stygia* Hebard, 1926	o				o	o	o	o	(Bonfils 1975; Bruijning 1959; Hebard 1926; Pellens and Grandcolas 2008; Perez 1988; Princis 1963; Rocha e Silva Albuquerque and Gurney 1962)
*Pseudomops affinis* (Burmeister, 1838)					o	o	o	o	(Bruijning 1959; Hebard 1926, 1929; Pellens and Grandcolas 2008; Princis 1963; Rocha e Silva Albuquerque and Gurney 1962; Walker 1868)
*Pseudomops angustus* Walker, 1868	o								(Bonfils 1987; Perez 1988; Princis 1963)
*Pseudomops brunneri* (Saussure, 1869)					o	o			(Bruijning 1959; Hebard 1929; Princis 1963)
*Pseudomops crinicornis* (Burmeister, 1838)					o				(Rehn 1906)
*Pseudomops luctuosus* (Saussure, 1868)					o	o	o		(Bruijning 1959; Hebard 1926; Princis 1963)
*Pseudomops oblongatus* (Linnaeus, 1758)					?	o	o		(Bonfils 1975; Bruijning 1959; Princis 1963; Walker 1868)
*Xestoblatta agautierae* Grandcolas, 1992					+		o		(Grandcolas 1992a)
*Xestoblatta amaparica* Rocha e Silva Albuquerque & Gurney, 1962	o							o	(Bonfils 1987; Pellens and Grandcolas 2008; Perez 1988; Princis 1963; Rocha e Silva Albuquerque and Gurney 1962)
*Xestoblatta carbuncula* Grandcolas, 1992							o		(Grandcolas 1992a)
*Xestoblatta castanea* Hebard, 1926						o	o		(Bruijning 1959; Hebard 1926; Princis 1963)
*Xestoblatta cavicola* Grandcolas, 1992							o		(Grandcolas 1992a)
*Xestoblatta jygautieri* Grandcolas, 1992							o		(Grandcolas 1992a)
*Xestoblatta micra* Hebard, 1921					o				(Princis 1963)
*Xestoblatta nourragui* Grandcolas, 1992							o		(Grandcolas 1992a)
*Xestoblatta nyctiboroides* (Rehn, 1906)					o		o		(Bruijning 1959; Hebard 1926; Princis 1963; Rehn 1906)
*Xestoblatta berenbaumae* sp. n.					+				New record
*Xestoblatta surinamensis* Bruijning, 1959						o	o		(Bonfils 1975; Bruijning 1959; Princis 1963)
**Nyctiborinae**									
*Megaloblatta insignis* (Serville, 1838)						o	o		(Bruijning 1959; Hebard 1926; Princis 1963)
*Nyctibora brunnea* (Thunberg, 1826)	o						o		(Bonfils 1975; Perez 1988; Hebard 1921b)
*Nyctibora dichropoda* Hebard, 1926					+	o	o		(Hebard 1926; Princis 1963)
*Nyctibora latipennis* Burmeister, 1838					o	o	o		(Bruijning 1959; Hebard 1926; Princis 1963; Rehn 1906; Walker 1868)
*Nyctibora tenebrosa* Walker, 1868					o	o	o	o	(Bonfils 1975 1987; Bruijning 1959; Hebard 1926; Pellens and Grandcolas 2008; Princis 1963; Walker 1868)
*Paramuzoa alsopi* Grandcolas, 1993							o		(Grandcolas 1993a)
*Paratropes elegans* (Burmeister, 1838)					o	o	o	o	(Bruijning 1959; Hebard 1921b; Pellens and Grandcolas 2008; Princis 1963; Rehn 1906; Rocha e Silva Albuquerque and Gurney 1962; Walker 1868)
*Paratropes phalerata* (Erichson, 1848)					o	o	o		(Bruijning 1959; Hebard 1929; Princis 1963; Princis and Kevan 1955)
*Pseudischnoptera lineata* (Olivier, 1789)						o	o		(Princis 1963; Hebard 1921b, 1926; Rehn and Hebard 1927)
**Pseudophyllodromiinae**									
*Amazonina conspersa* (Brunner von Wattenwyl, 1865)	o				o	o	o	o	(Bruijning 1959; Hebard 1926; Pellens and Grandcolas 2008; Perez 1988; Princis 1963)
*Amazonina impuctata* Rocha e Silva Albuquerque, 1995	o								(Bonfils 1987; Perez 1988; Princis 1963)
*Amazonina lanei* Rocha e Silva Albuquerque, 1962	o							o	(Pellens and Grandcolas 2008; Perez 1988; Rocha e Silva Albuquerque and Gurney 1962)
*Amazonina platystylata* (Hebard, 1921)					o	o	o		(Bruijning 1959; Perez 1988; Hebard 1921b, 1929)
*Arawakina frontalis* Hebard, 1926					o		o		(Bruijning 1959; Hebard 1926; Princis 1963)
*Calhypnorna* sp. Saussure & Zehntner, 1893					+				New record
*Cariblatta personata* Rehn, 1916							o		(Bruijning 1959; Hebard 1926)
*Cariblattoides gruneri* Bonfils, 1975							o		(Bonfils 1975)
*Cariblattoides guyanensis* Bonfils, 1975							o		(Bonfils 1975)
*Cariblattoides sinnamariensis* Bonfils, 1975							o		(Bonfils 1975)
*Ceratinoptera picta* Brunner von Wattenwyl, 1865						o	o		(Bonfils 1975; Bruijning 1959; Hebard 1926; Princis 1963; Princis and Kevan 1955)
*Ceratinoptera albonervosa* Rehn, 1916						o	o		(Bonfils 1975; Bruijning 1959; Hebard 1926; Princis 1963)
*Ceratinoptera barticae* Hebard, 1921					o	o	o		(Bonfils 1975; Bruijning 1959; Hebard 1921b, 1926; Princis 1963)
*Ceratinoptera cistelina* (Walker, 1868)						o	o		(Bonfils 1975; Bruijning 1959; Hebard 1926)
*Ceratinoptera elegantula* Hebard, 1926						o	o		(Bruijning 1959; Hebard 1926; Princis 1963)
*Ceratinoptera fuscipennis* Hebard, 1920						o	o		(Bruijning 1959; Hebard 1926; Princis 1963)
*Ceratinoptera galibi* Hebard, 1926						o	o		(Bonfils 1975; Bruijning 1959)
*Ceratinoptera gatunae* Hebard, 1921						o	o		(Bruijning 1959; Hebard 1926; Perez 1988; Princis 1963)
*Ceratinoptera gracilis* (Saussure, 1862)					o	o			(Bruijning 1959; Princis 1963; Rehn 1906)
*Ceratinoptera guianae* Hebard, 1921					o	o	o		(Bruijning 1959; Hebard 1921b, 1926)
*Ceratinoptera heydei* Bruijning, 1959						o			(Bruijning 1959; Princis 1963)
*Ceratinoptera inversa* Hebard, 1926					o	o	o	o	(Bruijning 1959; Hebard 1926; Pellens and Grandcolas 2008; Princis 1963)
*Ceratinoptera lata* Rehn, 1916						o	o		(Bonfils 1975; Bruijning 1959; Hebard 1921b, 1926)
*Chorisoneura multivenosa* Saussure, 1869							o		(Beccaloni 2007)
*Chorisoneura parishi* Rehn, 1918					o	o	o		(Bruijning 1959; Hebard 1926; Perez 1988; Princis 1963)
*Chorisoneura splendida* Hebard, 1926					o	o	o		(Bonfils 1975; Bruijning 1959; Hebard 1926 1929; Princis 1963)
*Chorisoneura strigifrons* Hebard, 1926						o	o		(Bruijning 1959; Hebard 1926; Princis 1963)
*Chorisoneura stylata* Hebard, 1926					o	o			(Bruijning 1959; Hebard 1926; Princis 1963)
*Chorisoneura surinama* Saussure, 1868						o	o		(Bruijning 1959; Hebard 1926; Princis 1963)
*Chorisoneura vitrifera* (Walker, 1868)					o	o	o		(Bruijning 1959; Hebard 1926, 1929)
*Chorisoneura vivida* Rocha e Silva Albuquerque & Gurney, 1962								o	(Pellens and Grandcolas 2008; Rocha e Silva Albuquerque and Gurney 1962)
*Dendroblatta callizona* Rehn, 1928					o	o			(Princis 1963; Princis and Kevan 1955; Bruijning 1959; Rehn 1928)
*Dendroblatta cnephaia* Hebard, 1926						o	o		(Bruijning 1959; Hebard 1926; Princis 1963)
*Dendroblatta insignis* Hebard, 1926						o	o		(Bruijning 1959; Hebard 1926; Princis 1963)
*Dendroblatta coppenamensis* Bruijning, 1959						o			(Bruijning 1959; Princis 1963)
*Euphyllodromia atropos* Rehn, 1928					o	o	o		(Bonfils 1975; Bruijning 1959; Princis 1963; Rehn 1928)
*Euphyllodromia aurora* Rehn, 1932							o	o	(Bonfils 1975; Pellens and Grandcolas 2008; Rocha e Silva Albuquerque and Gurney 1962)
*Euphyllodromia chopardi* Hebard, 1921				o	o		o	o	(Bruijning 1959; Hebard 1921b; Pellens and Grandcolas 2008; Princis 1963; Rocha e Silva Albuquerque and Gurney 1962)
*Euphyllodromia elegans* (Shelford, 1907)							o	o	(Bonfils 1975; Bruijning 1959; Hebard 1926; Pellens and Grandcolas 2008; Rehn 1928; Rocha e Silva Albuquerque and Gurney 1962)
*Euphyllodromia fasciatella* (Saussure, 1868)					o	o			(Bruijning 1959; Hebard 1929; Princis 1963; Rehn 1906)
*Euphyllodromia hystrix* (Saussure, 1869)	?	?	?						(Bonfils 1987; Perez 1988; Princis 1963; Hebard 1929)
*Euphyllodromia literata* (Burmeister, 1838)					o	o	o	o	(Bruijning 1959; Hebard 1921b, 1926; Pellens and Grandcolas 2008; Princis 1963; Walker 1868)
*Euphyllodromia marowijnensis* Bruijning, 1959						o			(Bruijning 1959; Princis 1963)
*Euphyllodromia obscura* (Saussure, 1873)					o				(Rehn 1906)
*Euphyllodromia pavonacea* (Rehn, 1903)					o	o	o	o	(Bonfils 1975; Bruijning 1959; Pellens and Grandcolas 2008; Princis 1963; Rehn 1903, 1906; Rocha e Silva Albuquerque and Gurney 1962)
*Euphyllodromia prona* (Rehn, 1906)					o				(Bruijning 1959; Princis 1963; Rehn 1906)
*Euphyllodromia variegata* (Walker, 1868)						o	o	o	(Bruijning 1959; Hebard 1926; Pellens and Grandcolas 2008; Princis 1963; Rocha e Silva Albuquerque and Gurney 1962)
*Imblattella litosoma* (Hebard, 1926)						o	o	o	(Bonfils 1975; Bruijning 1959; Hebard 1926; Pellens and Grandcolas 2008; Princis 1963; Rocha e Silva Albuquerque and Gurney 1962)
*Leuropeltis atopa* Hebard, 1921							o		(Bruijning 1959; Hebard 1921b 1926)
*Leuropeltis gurneyi* Rocha e Silva Albuquerque, 1964	o								(Bonfils 1987; Perez 1988; Princis 1963)
*Lophoblatta arawaka* Hebard, 1929					o	o			(Bonfils 1975, 1987; Bruijning 1959; Perez 1988; Princis 1963; Princis and Kevan 1955; Hebard 1929)
*Lophoblatta brevis* Rehn, 1937	o				o	o			(Bruijning 1959; Perez 1988; Princis 1963; Rehn 1937b)
*Lophoblatta pellucida* (Burmeister, 1838)						o	o	o	(Pellens and Grandcolas 2008; Princis 1963)
*Macrophyllodromia nigrigena* Hebard, 1926					o	o	o		(Bruijning 1959; Hebard 1926; Princis 1963)
*Nahublattella aristonice* Hebard, 1926						o	o		(Bonfils 1975; Hebard 1926; Princis 1963)
*Nahublattella incompta* (Hebard, 1926)							o		(Bonfils 1975; Bruijning 1959; Hebard 1926)
*Neoblattella adspersicollis* (Stål, 1860)								o	(Bruijning 1959; Hebard 1921b; Pellens and Grandcolas 2008; Rocha e Silva Albuquerque and Gurney 1962)
*Neoblattella binodosa* Hebard, 1926						o	o		(Bruijning 1959; Hebard 1926; Princis 1963)
*Neoblattella elegantula* Rocha e Silva Albuquerque, 1964	o								(Perez 1988; Princis 1963; Lopes and de Oliveira 2004)
*Neoblattella guianae* Hebard, 1929					o		o	o	(Bonfils 1975; Hebard 1929; Lopes and de Oliveira 2004; Pellens and Grandcolas 2008; Rocha e Silva Albuquerque and Gurney 1962)
*Neoblattella longior* Hebard, 1926						o	o		(Bruijning 1959; Hebard 1926; Lopes and de Oliveira 2004; Princis 1963)
*Neoblattella nodipennis* Hebard						o	o		(Bruijning 1959; Hebard 1926; Princis 1963)
*Neoblattella picta* Rocha e Silva Albuquerque & Gurney, 1962								o	(Pellens and Grandcolas 2008; Rocha e Silva Albuquerque and Gurney 1962)
*Neoblattella poecilops* Hebard, 1926						o	o		(Bruijning 1959; Hebard 1926; Lopes and de Oliveira 2004; Princis 1963)
*Neoblattella titania* (Rehn, 1903)					o	o	o		(Bruijning 1959; Hebard 1926; Princis 1963; Rehn 1903)
*Neoblattella unifascia* Hebard, 1926							o		(Bruijning 1959; Hebard 1926; Lopes and de Oliveira 2004; Princis 1963)
*Plectoptera pulicaria* Saussure & Zehntner, 1893						o	o		(Bruijning 1959; Hebard 1926; Princis 1963)
*Riatia distincta* (Hebard, 1926)						o	o		(Bruijning 1959; Hebard 1926)
*Riatia fulgida* (Saussure, 1862)					o	o			(Bruijning 1959; Rehn 1906; Princis 1963)
*Riatia orientis* (Hebard, 1926)	o				o	o	o		(Bruijning 1959; Hebard 1926; Princis 1963; Perez 1988; Princis and Kevan 1955)
*Riatia stylata* (Hebard, 1926)						o	o		(Bonfils 1975; Bruijning 1959; Hebard 1926)
*Riatia variegata* Rocha e Silva Albuquerque & Aguiar, 1976	o								(Perez 1988)
*Riatia venezuelana* Rocha e Silva Albuquerque, 1964	o								(Perez 1988; Princis 1963)
*Sciablatta galibi* Hebard, 1926							o		(Bruijning 1959; Hebard 1926)
*Sciablatta poecila* Hebard, 1921					o		o		(Bruijning 1959; Hebard 1921b; Princis 1963)
*Supella longipalpa* (Fabricius, 1798)	o	o	o	o	o	o	o	o	(Bonfils 1987; Bruijning 1959; Hebard 1929; Perez 1988; Princis 1963; Princis and Kevan 1955)
*Tairella carinatifrons* Hebard, 1926						o	o		(Hebard 1926; Princis 1963)
*Trioblattella callosoma* (Hebard, 1926)					o		o	o	(Bonfils 1975; Bruijning 1959; Hebard 1926; Pellens and Grandcolas 2008; Princis 1963; Rocha e Silva Albuquerque and Gurney 1962)
**Lamproblattidae**									
**Lamproblattinae**									
*Lamproblatta albipalpus* Hebard, 1919	?	?	?					o	(Hebard 1931; Pellens and Grandcolas 2008; Princis and Kevan 1955; Rehn 1930; Rocha e Silva Albuquerque and Gurney 1962)
*Lamproblatta ancistroides* Rehn, 1930	?	?	?						(Perez 1988; Princis 1963)

**Table 2.** Allometry of new records of cockroaches from Guyana reported in the text. All values are lengths reported in millimeters. NA – refers to specimens which are damaged and therefore cannot be measured or refer to specimens for which the listed measurement does not apply. Specimens with asymmetrical styli have lengths of both right (R.) and left (L.) styli given. When possible, broken specimens had relevant measurements estimated (est.) by piecing together damaged parts or extrapolating visually.

Morphological feature			*Eublaberus distanti*	*Eublaberus* sp.		*Neorhicnoda maronensis*	*Colapteroblatta surinama*		*Epilampra opaca*		*Epilampra sodalis*	*Thanatophyllum akinetum*
			Adult ♂	Adult ♂	Adult ♂	Adult ♂	Adult ♂	Adult ♀	Adult ♂	Adult ♀	Adult ♀	Adult ♂
			DEKBO0843	DEKBO0842	DEKBO0844	DECBA0615	DECBA0703	DECBA1810	DECBA1845	DECBA1847	DECBA0401	DECBA0611
Head	Greatest width		6.5	6.5	6.8	4.5	3.0	3.4	3.3	3.8	5.0	3.8
	Medial length		7.5	7.0	7.1	5.4	3.1	3.4	3.1	4.5	5.5	3.8
Pronotum	Greatest width		17.5	15.5	15.0	12.9	6.0	6.5	6.0	8.0	10.0	10.0
	Medial length		11.0	10.5	10.0	8.8	4.7	4.5	4.6	6.5	7.8	7.0
Leg	Front	Femur	6.0	6.0	5.7	5.0	2.2	2.2	3.0	3.5	4.8	4.0
		Tibia	2.8	4.5	3.9	2.2	1.4	1.6	2.0	2.5	2.5	2.2
	Middle	Femur	9.5	8.3	8.0	6.3	2.3	2.7	4.5	5.5	5.6	5.0
		Tibia	7.0	7.0	6.0	4.5	1.8	1.9	4.0	4.0	5.5	4.9
	Hind	Femur	10.0	9.0	8.0	6.7	3.1	2.7	5.8	6.0	7.0	5.8
		Tibia	13.0	11.0	10.0	8.5	3.7	3.4	7.9	9.2	10.0	8.0
Cerci length			3.0	3.3	2.8	1.5	0.6	0.5	3.0	2.2	3.3	1.2
Styli length			0.8	0.8	1.0	0.5	0.3	NA	0.5	NA	NA	NA
Tegminal length			39.5	NA	NA	NA	10.0	2.0	20.0	24.5	28.0 (est.)	22.0
Total body length			43.5	44.0	46.0	34.0	15.7	19.3	20.5	25.0	31.0	26.0
Morphological feature			*Anaplecta parviceps*	*Anisopygia decora*	*Ischnoptera atrata*		*Xestoblatta agautierae*		*Nyctibora dichropoda*	*Chorisoneura inversa*	*Dendroblatta callizona*	*Calhypnorna* sp.
			Adult ♂	Adult ♀	Adult ♂	Adult ♂	Adult ♂	Adult ♀	Adult ♂	Adult ♂	Adult ♀	Juvenile
			DECBA1843	DEKBO0504	DECBA2153	DEKBO0594	DEKBO0827	DEKBO0826	DECBA0302	DECBA1782	DECBA0805	DECBA1802
Head	Greatest width		1.0	1.8	3.1	3.5	2.6	2.4	4.9	1.6	2.4	1.5
	Medial length		1.1	1.9	3.8	4.2	3.3	3.0	6.0	1.5	2.8	1.3
Pronotum	Greatest width		1.6	3.9	5.9	6.7	5.0	5.3	11.0	2.9	4.9	1.7
	Medial length		1.1	2.4	4.0	4.8	3.4	3.8	6.0	1.8	3.0	1.6
Leg	Front	Femur	1.0	1.8	3.1	3.0	2.8	3.1	6.0	1.5	2.8	1.1
		Tibia	0.7	1.0	2.2	2.0	1.7	2.0	4.0	1.0	1.9	0.7
	Middle	Femur	1.4	2.2	4.0	3.8	3.5	4.0	7.6	?	3.4	1.4
		Tibia	1.2	1.8	4.1	3.6	3.4	3.5	7.0	?	2.8	1.0
	Hind	Femur	NA	2.5	5.2	4.3	4.0	4.6	9.0	2.3	4.3	1.4
		Tibia	NA	2.9	6.0	6.1	6.0	5.1	12.0	2.7	4.6	1.3
Cerci length			NA	1.6	3.6	3.0	2.3	2.3	7.0	1.6	3.3	0.6
Styli length			0.1	NA	0.6 (L.) 0.9 (R.)	0.5 (L.) 0.7 (R.)	NA	NA	2.0 (L.) 1.2 (R.)	0.6	NA	0.2
Tegminal length			3.7	1.3	22.0	21.8	10.0	10.0	36.0	7.4	9.8	NA
Total body length			4.7	8.9	21.8	21.3	17.0 (est.)	15.0	37.0	7.8	13.8	7.3

### Subfamily: Blaberinae

#### Eublaberus distanti

Taxon classificationAnimaliaBlattodeaBlaberidae

(Kirby, 1903)

##### Materials.

*Adult* ♂.

Voucher number: DEKBO0843.

Collection locale. Karanambu Ranch, Rupununi, Guyana.

GPS: 3°45'2.2"N, 59°18'31.2"W.

Date: 7 – June – 2013.

Collectors. Dominic A. Evangelista, Oswin Ambrose, Susan George, and Megan M. Wilson.

##### Collection/ecological information.

This specimen was collected in the bathroom of one of the cabins at the camp of Karanambu Ranch.

##### Known geographic distribution.

Guatemala, Costa Rica, Panama, Colombia, Trinidad and Tobago, French Guiana, Suriname, Guyana and Brazil

#### Neorhicnoda maronensis

Taxon classificationAnimaliaBlattodeaBlaberidae

(Hebard, 1921)

##### Materials.

*Adult* ♂ Figure 1.

Voucher number: DECBA0615.

GenBank accession number: KF155090.

Collection locale. CEIBA Biological Station, Madewini, Guyana.

GPS: 6°29'N, 58°13'W.

Date: 02 – January – 2012.

Collectors. Dominic A. Evangelista, Ian Biazzo, Joseph A. Evangelista, Paul Frandsen, William R. Kuhn, and Jessica L. Ware.

##### Collection/ecological information.

This specimen was caught in a pitfall trap baited with beer in an uplands secondary forest.

##### Morphological identification.

This specimen agrees with the description of the male genitalia in Grandcolas (1992b).

##### Known geographic distribution.

Guyana (new record), Suriname, and French Guiana.

**Figure 1.**
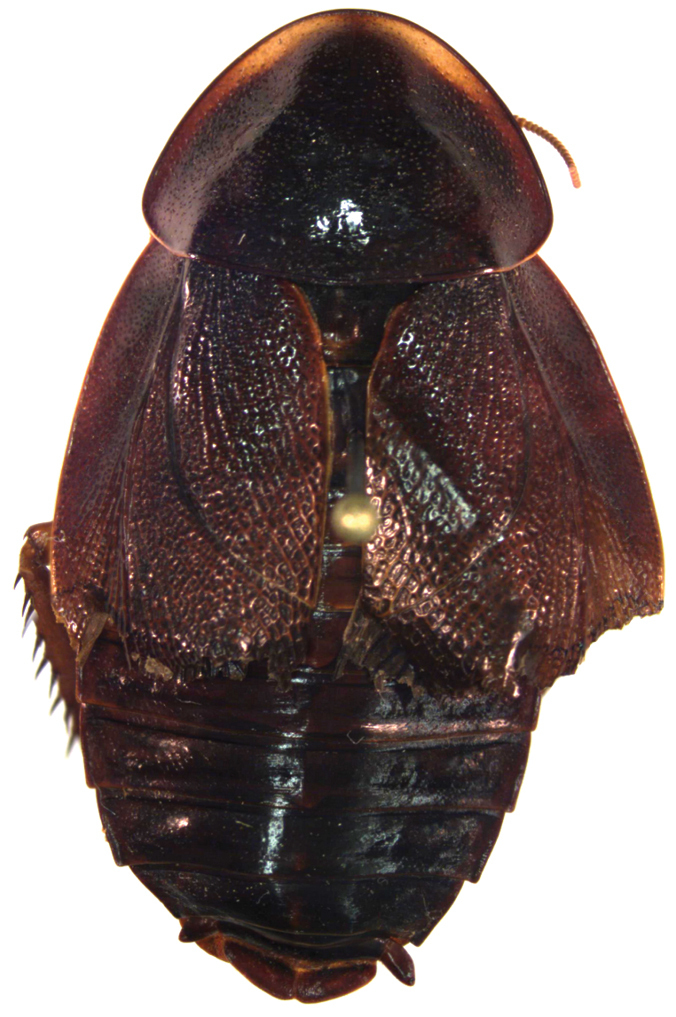
*Neorhicnoda maronensis* adult male (DECBA0615).

### Subfamily: Epilamprinae

#### Colapteroblatta surinama

Taxon classificationAnimaliaBlattodeaBlaberidae

(Saussure, 1868)

##### Materials.

**Figure 2.**
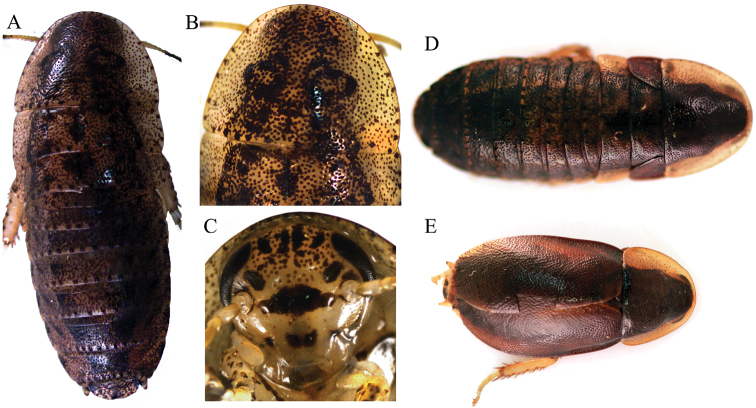
*Colapteroblatta surinama*. **A–C** Juvenile (dorsal aspect, pronotum, ventral aspect of head) **D** Adult female, dorsal aspect **E** Adult male, dorsal aspect. Photos not to scale.

*Adult* ♂ Figure 2E.

Voucher number: DECBA0703.

GenBank accession number: KF155029.

Collection locale. CEIBA Biological Station, Madewini, Guyana.

GPS: 6°29'N, 58°13'W.

Date: 05 – August – 2011.

Collectors. Dominic A. Evangelista, Ian Biazzo, Manpreet K. Kohli, Melissa Sanchez-Herrera, Nicole Sroczinski, and Jessica L. Ware.

##### Collection/ecological information.

This specimen was collected in an uplands secondary forest from within a rotting vine.

##### Morphological identification.

This specimen was identified using Roth and Gutierrez (1998).

*Adult* ♀ Figure 2D.

Voucher number: DECBA1810.

GenBank accession number: KF155126.

Collection locale. CEIBA Biological Station, Madewini, Guyana.

GPS: 6°29'N, 58°13'W.

Date: 20 – August – 2011.

Collectors. Dominic Evangelista and William R. Kuhn.

##### Collection/ecological information.

This specimen was collected in an uplands secondary forest from within an arboreal bromeliad.

##### Morphological identification.

This specimen was identified using Roth and Gutierrez (1998).

*Juvenile* Figure 2A–C.

Voucher number: DECBA1811.

GenBank accession number: KF155112.

Collection locale. CEIBA Biological Station, Madewini, Guyana.

GPS: 6°29'N, 58°13'W.

Date: 17 – August – 2013.

Collectors. Dominic Evangelista and William R. Kuhn.

##### Collection/ecological information.

This specimen was collected on vegetation in an uplands secondary forest.

##### Morphological identification.

This specimen was associated to its adult morph using barcodes in Evangelista et al. (2014). The overall coloration of the juvenile specimens of this species is more similar to that of *Colapteroblatta darlingtoni* Roth & Gutiérrez, 1998 and *Colapteroblatta rehni* Roth & Gutiérrez, 1998 than to that of the adults of its own species (see Figure 2).

**Genetic information and evolutionary placement.** All three specimens have nearly identical cytochrome oxidase I (COI) haplotypes but their position could not be determined relative to other cockroach species with the data evaluated by Evangelista et al. (2014).

##### Known geographic distribution.

Guyana, Suriname.

#### Epilampra opaca

Taxon classificationAnimaliaBlattodeaBlaberidae

Walker, 1868

##### Materials.

**Figure 3.**
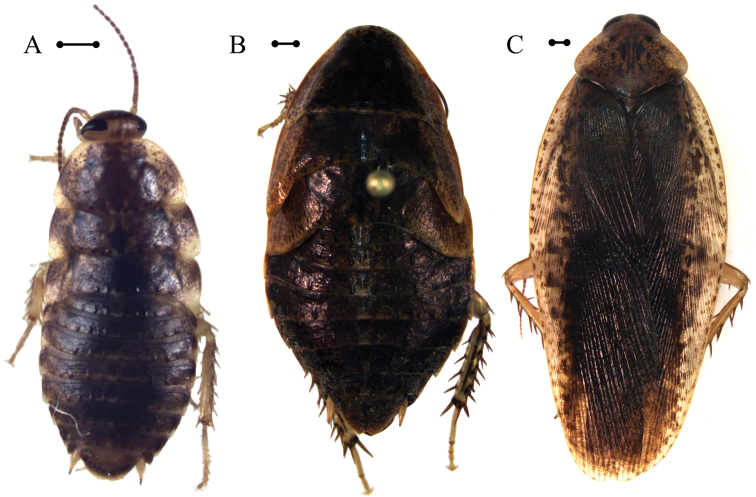
*Epilampra opaca*. **A** Early juvenile instar (DEDSM0141) **B** Late juvenile instar (DECBA1706) **C** Adult (DECBA1845). Scale bars approximate 1 mm.

*Adult* ♂ Figure 3B.

Voucher number: DECBA1845.

GenBank accession number: KF155125.

Collection locale. CEIBA Biological Station, Madewini, Guyana.

GPS: 6°29'N, 58°13'W.

Date: 18 – August – 2012.

Collectors. Dominic A. Evangelista and William R. Kuhn.

*Adult* ♀

Voucher number: DECBA1847.

GenBank accession number: KF155124.

Collection locale. CEIBA Biological Station, Madewini, Guyana.

GPS: 6°29"N, 58°13"W.

Date: 5 – August – 2011.

Collectors. Dominic A. Evangelista, Ian Biazzo, Manpreet K. Kohli, Melissa Sanchez-Herrera, Nicole Sroczinski and Jessica L. Ware.

##### Collection/ecological information.

The adult male (DECBA1845) was collected at a light trap. Adult female (DECBA1847) was collected by hand in the leaf litter by a small pond. Most late instar individuals of this species were also collected at the edge of this pond and some were collected in pitfall traps baited with beer. Early instar individuals of this species were collected from within bromeliads.

##### Genetic information.

The two adult specimens reported here, as well as three juvenile individuals (Voucher and accession numbers: DEDSM0141 – KF155097, DECBA1706 – KF155089, DECBA0205 – KF155088) have identical COI barcodes and are sister to each other on the tree. However, other individuals (similar to *Epilampra opaca*) included in the analysis (Voucher and accession numbers: DECBA0214 – KF155018, DECBA0216 – KF155017, DECBA0606 – KF155013, DECBA1101 – KF155016, DECBA0605 – KF155012, DECBA0608 – KF155015) are more genetically diverse and are only supported as monophyletic by 63% bootstrap support.

##### Morphological identification.

There is a great deal of intraspecific variation in the morphology of this species. Early instar nymphs are difficult to associate to later instar nymphs, all of which are entirely unrecognizable from the adults (Figure 3A–C). Furthermore, there is variation within instars, where some later instar nymphs will appear to have a medially divided subgenital plate and others do not. This trait was not found to correlate with genetic differences (Evangelista et al. 2014).

The external morphology of this species provides little assistance in its identification, as most descriptions of it emphasize coloration that is both subtle and variable. However, the allometry of our specimens (Table 2) agree with those of Bruijning (1959). A definitive identification was made by comparison of genital morphology using Roth (1970b), particularly in the shape of the prepuce.

##### Known geographic distribution.

Venezuela (unverified), Guyana, Suriname, French Guiana and Brazil

**History and synonymy.** Walker (1868) first described both *Epilampra opaca* Walker, 1868 and *Epilampra substrigata* Walker, 1868. Hebard (1926) noted that *Epilampra opaca* Walker, 1868 has a highly variable morphology and may be synonymous with a few other *Epilampra* (e.g. *Epilampra conferta* Walker, 1868 syn. *stigmosa* Giglio-Tos, 1898, *Epilampra maculicollis* (Serville, 1838)). This variability is evident in the work published by Roth (1970b), which shows a great deal of variation in the genital morphology, in particular for L2d. Although it is not clear if anyone before Roth (1970b) examined the genitalia of these two species, both Shelford (1910) and Princis (1963) considered them to be synonyms. Roth’s (1970b) photos show that, although each species is intraspecifically variable, both are distinct and separable by the shape of L2d and the prepuce. Roth himself acknowledged this and considered the species as being separate. Although we have not examined any *Epilampra substrigata* Walker, 1868, we agree with Roth’s interpretation of the morphology and follow from his precedence in considering these separate (see Roth 1970b for the opinions of Princis and Gurney on the status of these two species).

#### Epilampra sodalis

Taxon classificationAnimaliaBlattodeaBlaberidae

Walker, 1868

##### Materials.

**Figure 4.**
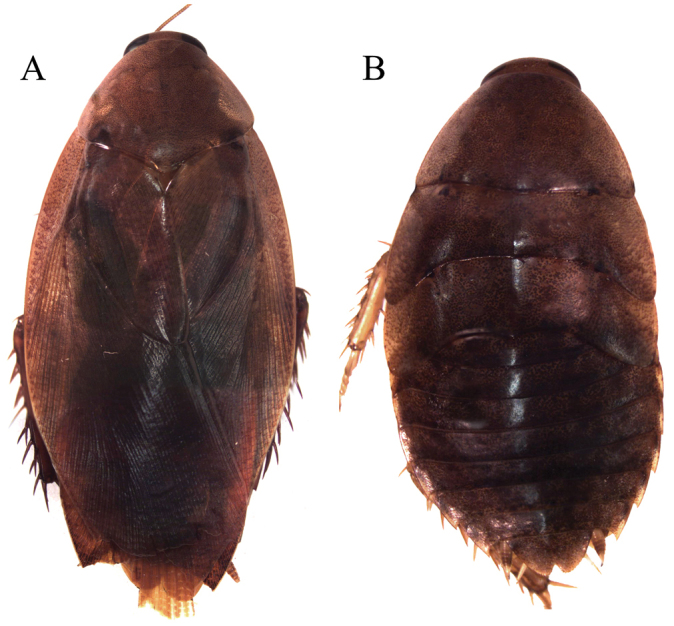
*Epilampra sodali*. **A** Adult male dorsal view (DECBA0401) **B** Juvenile dorsal view (DECBA2163). Add scale bar.

*Adult* ♂ Figure 4A.

Voucher number: DECBA0401.

GenBank accession number: KF155063.

Collection locale. CEIBA Biological Station, Madewini, Guyana.

GPS: 6°29'N, 58°13'W.

Date: 31 – July to 6 – August – 2011.

Collectors. Dominic A. Evangelista, Ian Biazzo, Manpreet K. Kohli, Melissa Sanchez-Herrera, Nicole Sroczinski, and Jessica L. Ware.

##### Collection/ecological information.

This specimen was collected at a light trap.

##### Morphological identification.

This specimen agrees with the description the synonym *Epilampra cinnamomea* (Hebard 1926).

Juvenile

Voucher number: DECBA1702.

GenBank accession number: KF155068.

Collection locale. CEIBA Biological Station, Madewini, Guyana.

GPS: 6°29'N, 58°13'W.

Date: 27 – December – 2011.

Collectors. Dominic A. Evangelista, Ian Biazzo, Joseph A. Evangelista, Paul Frandsen, William R. Kuhn and Jessica L. Ware.

Juvenile

Voucher number: DECBA1701.

GenBank accession number: KF155069.

Collection locale. CEIBA Biological Station, Madewini, Guyana.

GPS: 6°29'N, 58°13'W.

Date: 10 – January – 2012.

Collectors. Dominic A. Evangelista, Ian Biazzo, Joseph A. Evangelista, Paul Frandsen, William R. Kuhn and Jessica L. Ware.

##### Collection/ecological information.

Both of these juvenile specimens were collected at the edge of a small pond.

**Genetic information and evolutionary placement.** These three specimens (previous reported as “Blaberidae sp. 04”) were placed in the same clade with 90% bootstrap support.

##### Known geographic distribution.

Venezuela, Guyana (new record), Suriname, French Guiana and Brazil

### Subfamily: Zetoborinae

#### Thanatophyllum akinetum

Taxon classificationAnimaliaBlattodeaBlaberidae

Grandcolas, 1991

##### Materials.

*Adult* ♂ Figure 5.

Voucher number: DECBA0611.

GenBank accession number: KF155066.

Collection locale. CEIBA Biological Station, Madewini, Guyana.

GPS: 6°29'N, 58°13'W.

Date: 28 – December – 2011.

Collectors. Dominic A. Evangelista, Ian Biazzo, Joseph A. Evangelista, Paul Frandsen, William R. Kuhn and Jessica L. Ware.

##### Collection/ecological information.

This specimen was collected by hand on vegetation in an uplands secondary forest.

##### Morphological identification.

This specimen agrees with the description of the head and male genitalia of Grandcolas (1990).

##### Known geographic distribution.

Guyana (new record) and French Guiana.

**Figure 5.**
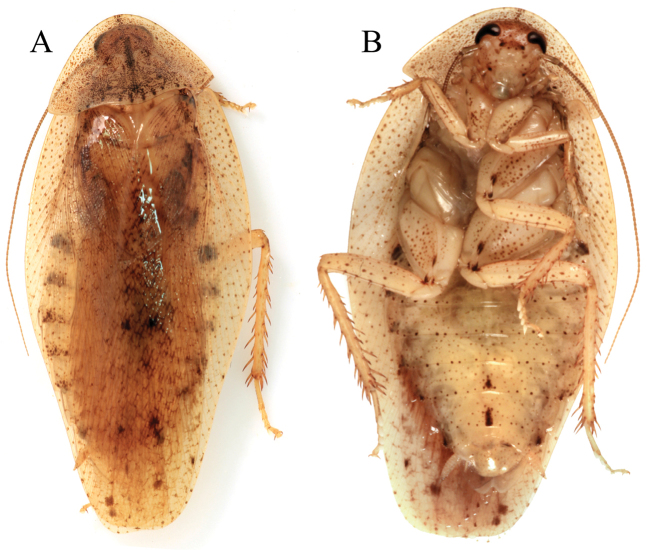
*Thanatophyllum akinetum* adult male (DECBA0611). **A** Dorsal view **B** Ventral view.

### Family: “Ectobiidae”

#### Subfamily: Anaplectinae

##### Anaplecta parviceps

Taxon classificationAnimaliaBlattodeaEctobiidae

(Walker, 1868)

###### Materials.

*Adult* ♂ Figure 6.

Voucher number: DECBA1843.

GenBank accession number: KF155137.

Collection locale. CEIBA Biological Station, Madewini, Guyana.

GPS: 6°29'N, 58°13'W.

Date: 16 – August – 2012.

Collectors. Dominic A. Evangelista and William R. Kuhn.

###### Collection/ecological information.

This specimen and another adult male (Voucher number: DECBA1841) were collected at a light trap near the camp of CEIBA Biological Station on the date noted above. A juvenile of this species was also collected at the same locale, found crawling through a benab between 21 and 24 of August 2012 (Voucher number: DECBA1842).

###### Morphological identification.

The specimen agrees with the description of the synonym *Anaplecta insignis* of Hebard (1926). Other specimens were identified by comparison with specimen DECBA1843.

**Genetic information and evolutionary placement.** The COI barcodes of this specimen (previously reported as “Blattodea sp. 18”) falls sister to another specimen identified as *Anaplecta* sp. (previously reported as “Ectobiidae sp. 04”; Voucher number: DEDSM0111; GenBank accession number: KF155041) but with 25% bootstrap support. This other species is not reported in this paper due to an uncertainty in specific identification.

###### Known geographic distribution.

Guyana, Suriname, French Guiana, Brazil (Rio de Janeiro), Brazil (Pará), and Brazil (Amapá).

**Figure 6.**
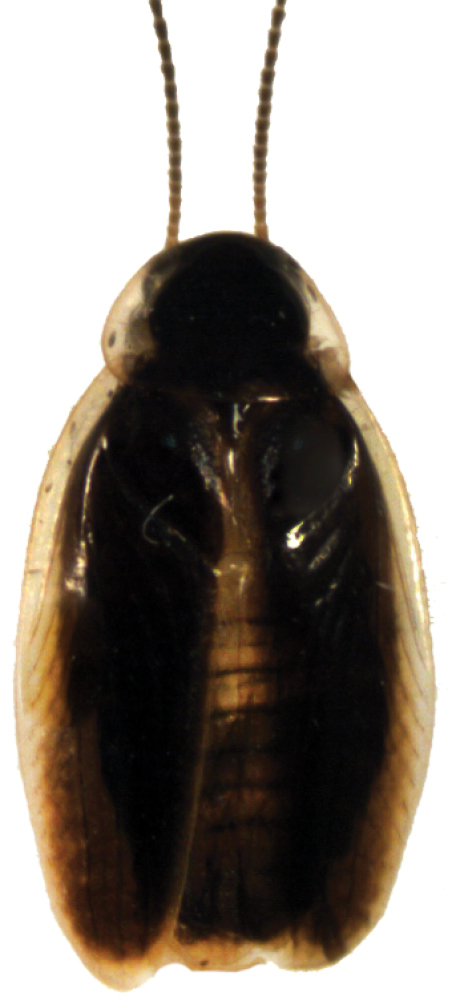
*Anaplecta parviceps* adult male (DECBA1843).

#### Subfamily: Blattellinae

##### Anisopygia decora

Taxon classificationAnimaliaBlattodeaEctobiidae

Hebard, 1926

###### Materials.

*Adult* ♀ Figure 7.

Voucher number: DEKBO0504.

Collection locale. Capuchin Trail, Karanambo Ranch, Rupununi, Guyana.

GPS: 3°44'43.70"N, 59°18'51.88"W.

Date: 10 – June – 2013.

Collectors. Dominic A. Evangelista, Oswin Ambrose, Susan George, and Megan M. Wilson.

###### Collection/ecological information.

This specimen was collected by hand in an undisturbed forested area. This is the first record of this specimen from Guyana.

###### Morphological identification.

This specimen was identified by comparison with Hebard’s description (1926).

###### Known geographic distribution.

Guyana (new record) and French Guiana.

**Figure 7.**
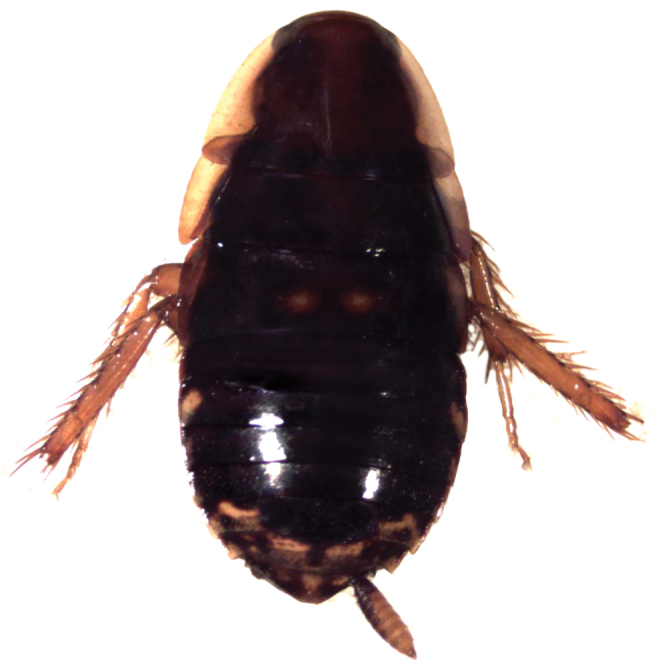
*Anisopygia decora* adult female (DEKBO0504).

##### Ischnoptera atrata

Taxon classificationAnimaliaBlattodeaEctobiidae

Hebard, 1916

###### Materials.

*Adult* ♂.

Voucher number: DECBA2153.

Collection locale. CEIBA Biological Station, Madewini, Guyana.

GPS: 6°29'N, 58°13'W.

Collection date: December – 2011.

Collectors. Dominic Evangelista, Ian Biazzo, Joseph A. Evangelista, Paul Frandsen, William R. Kuhn, and Jessica L. Ware.

###### Collection/ecological information.

This specimen was collected in a pitfall trap baited with beer in an uplands secondary forest area.

*Adult* ♂ Figure 8.

Voucher number: DEKBO0594.

Collection locale. Karanamabu Ranch, Rupununi, Guyana.

GPS: 3°45'0.1"N, 59°18'53.7"W.

Collection date: 10 – June – 2013.

Collectors. Dominic A. Evangelista, Oswin Ambrose, Susan George, and Megan M. Wilson.

###### Collection/ecological information.

This specimen was collected in a pitfall trap baited with beer in a forest proximal to the Rupununi River.

###### Morphological identification.

Both specimens mostly agree with the description and figures of Hebard (1916). However, there are slight differences in the supra-anal plate when compared to Hebard’s illustration. The white region on the SA plate of our specimen is slightly larger than in Hebard’s illustration. It is possible that this is a different species than that described by Hebard, but this cannot be fully determined without a full phylogenetic treatment of sexual morphology and genetic information of individuals from both Trinidad and Guyana.

###### Known geographic distribution.

Trinidad and Tobago, and Guyana

**Figure 8.**
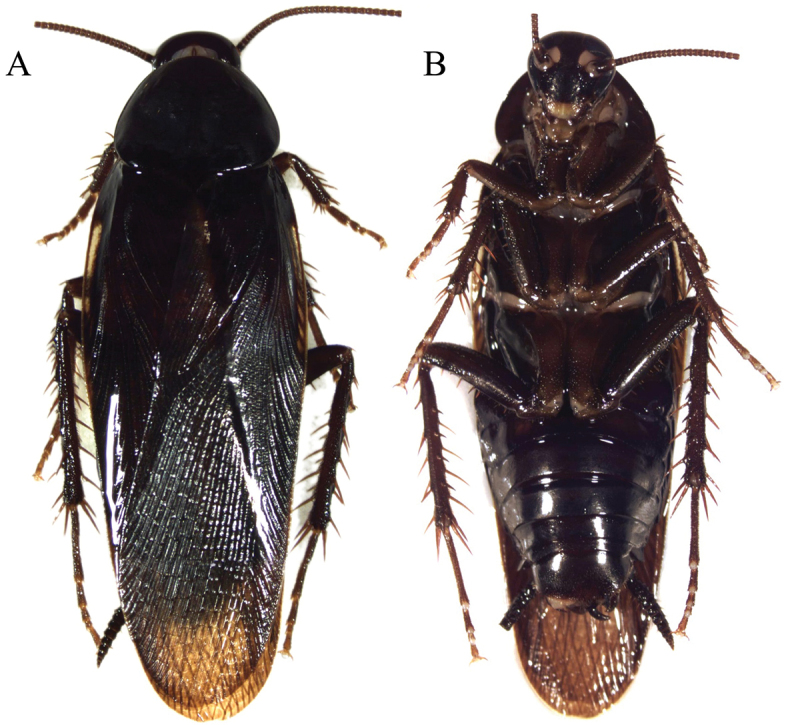
*Ischnoptera atrata* adult male (DEKBO0594). **A** Dorsal view **B** Ventral view.

##### Xestoblatta berenbaumae

Taxon classificationAnimaliaBlattodeaEctobiidae

Evangelista, Kaplan, & Ware sp. n.

http://zoobank.org/0DCFF043-F783-49E4-8576-4A2AD402AF82

###### Authors of description.

Evangelista, Kaplan, & Ware.

###### Holotype.

**Figure 9.**
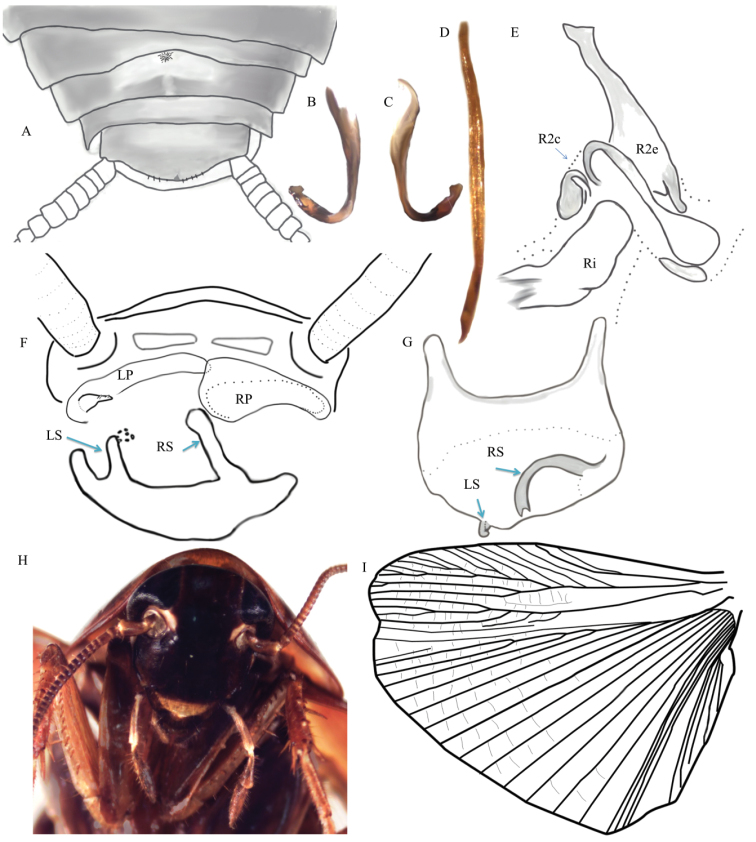
*Xestoblatta berenbaumae* sp. n. **A** Dorsal view of abdomen showing the simple tergal gland (DECBA2023) **B, C** Hooked left phallomere **D** Ventral medial phallomere (L2vm) **E** Right phallomere. R2e – external sclerite, R2i – internal sclerite, R2c – cleft sclerite **F** Posterior view of abdomen showing paraprocts and subgenital plate. RS-right stylus, LS-left stylus with small translucent ball at tip, LP-left paraproct reduced and specialized with polydentate spine, RP-unspecialized right paraproct. Illustration is a composite of multiple individuals **G** Dorsal view of sub-genital plate (DECBA1967) **H** Head of adult male **I** Hindwing (DECBA0801). Photos and illustrations contributed by Kayla Kaplan and Dominic A. Evangelista.

*Adult* ♂ Figure 9B–E, G.

Voucher number: DECBA2109.

Type locality. CEIBA Biological Station, Madewini, Guyana.

GPS: 6°29'N, 58°13'W.

Collection date: 17 to 18 – August – 2012.

Collectors. Dominic A. Evangelista and William R. Kuhn.

###### Type information.

The holotype specimen is stored in ethanol with genitalia in a separate ethanol vial and is deposited at the Center for Biodiversity at the University of Guyana.

###### Collection/ecological information.

This specimen was collected in a pitfall trap baited with beer and fruit in an uplands secondary forest in CEIBA Biological Station.

###### Morphological identification.

This specimen was identified as *Xestoblatta* Hebard, 1916 by the position of the hooked phallomere (left), the presence of the external modification of the tergum as part of the dorsal tergal gland (Figure 9A), incomplete rami on the ulnar vein of the hind wing (Figure 9I) and the spination (type A) on the ventro-anterior margin of the fore-femur.

###### Holotype morphological description.

Head uniformly colored a deep mahogany. Clypeus pale buffy. Ocellar spots easily distinguishable, smaller than antennal pits and white. Head otherwise without distinguishing features. Ocellar spots slightly closer together than eyes. Facial grooves on lateral most edge. See Figure 9H for a representative photo of the head.

**Figure 10.**
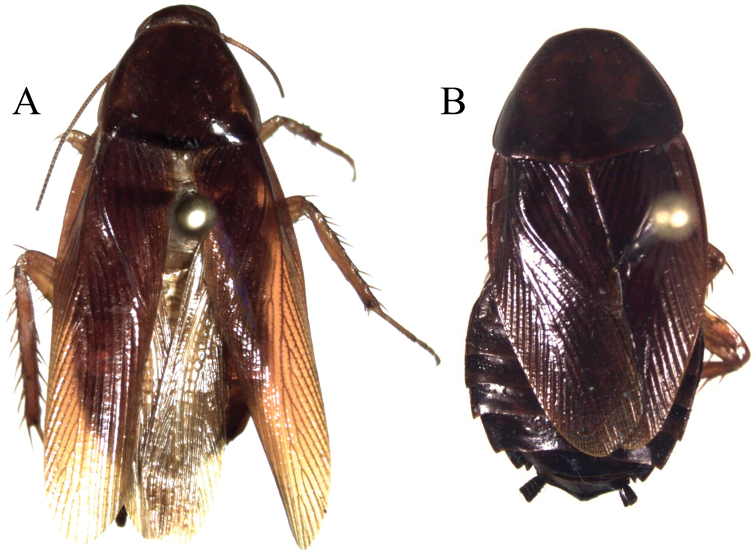
*Xestoblatta berenbaumae* sp. n. **A** Adult male dorsal view (DECBA2182) **B** Adult female dorsal view (DECBA2210).

Pronotum a uniformly reddish mahogany color (Figure 10A). Medial expansion on posterior margin of pronotum is barely noticeable. Ventral margin of pronotum not lined with hairs. Anterior margin of pronotum significantly conformed around the head. Leg coloration deep orange amber. Coxae with some diffuse black regions. Ventro-anterior margin of fore-femur with 14 (left) or 13 (right) spines decreasing in size from basal to apical, one slightly larger pre-apical spine and one large apical spine (16 total left, 15 total right). Ventro-posterior margin of forelimbs with 4 large spines and 1 apical spine. Ventro-anterior margin of middle leg has seven large spines and one apical spine. Middle leg also with one large genicular spine. Hind leg ventro-anterior margin has six spines, one apical spine, and one genicular spine. Pulvilli present on all tarsomeres. Arolia present but not surpassing the tips of the pretarsal claws. Claws symmetrical and unspecialized.

Ulnar vein with three incomplete rami and three complete rami (Figure 9I). Tegmina reddish mahogany with small patch of white under the base of the subcostal vein.

Supra-anal plate subtriangular with a blunt tip from dorsal view. Left paraproct modified into a tri-dentate spine (Figure 9F; bi-dentate in some other specimens). Sub-genital plate has both styli highly modified (Figure 9F, G). The right stylus is projecting dorso-medially from posterior margin, curving back posteriorly and terminating in a shape reminiscent of a bifurcated serpentine tongue. Left stylus projecting dorsally, shorter than right stylus and tipped with a small, translucent, irregularly shaped ball (Figure 9F, G).

Left phallomere (Figure 9B, C) hooked in apical third. (Hooked phallomere is about 1.5 mm long). Medial phallomere (Figure 9D) approximately three times the length of the left phallomere, roughly uniform width, and a slight slender curve in the posterior end. R2c (Figure 9E) divided into two sclerites that form dual concave cups that meet dorsally.

Dorsal modification of terga as part of the dorsal tergal gland. Modification represented by a small patch of hairs with a concave semi-circular modification of the margin of the segment anterior to the gland. See Figure 9A for an illustration of a representative dorsum.

Medium sized hairs (~ 2 mm) covering entire body roughly uniformly, yet sparsely.

###### Other adult male paratypes.

Voucher numbers: DECBA1967, DECBA0801, DECBA1958, DECBA2182, DECBA2092, DECBA2039

###### Collection/ecological information.

All additional male individuals reported here were collected in leaf litter pitfall traps baited with beer at various locations (dryer secondary uplands forest and wet primary lowlands forest) in CEIBA biological station.

###### Adult female paratype morphological description.

Voucher number: DECBA2074.

Head slightly darker in color than male with a more reflective surface. Other features of head similar to male.

Description of legs similar or identical to that of male with the following spination on the ventro-anterior margin of fore-femur: 13 (left) and 12 (right) spines decreasing in size from basal to apical, two larger preapical spines and one large apical spine (16 total left and 15 total right). Ventro-posterior margin of fore-femur four large spines and one apical spine. Ventro-anterior margin of mid-leg with seven large spines, one apical spine, and one genicular spine. Ventro-anterior margin of hind-leg with five large spines, one apical spine, and one genicular spine.

Tegmina and wings reduced and not reaching end of abdomen. Three incomplete and three complete rami on ulnar vein. Ulnar vein very faint in the reduced wings of the female (Figure 10B; Table 3).

Pronotum matches description of the male.

Subgenital plate slightly more abbreviated than in male. Paraprocts simple and unspecialized. Sub-genital plate simple and symmetrical.

###### Other adult female paratypes.

Voucher numbers: DECBA1787, DECBA1791, DECBA1792, and DECBA1793

###### Collection/ecological information.

All additional female individuals reported here were collected in leaf litter pitfall traps baited with beer in an uplands secondary forest at CEIBA biological station.

###### Summary of female morphology.

All individuals match the description of the above female and have the following spination on the vento-anterior margin of the fore-limb: 13 spines decreasing in size from basal to apical, one or two slightly larger preapical spines and one large apical spine making a total of 15 or 16 spines.

###### Juvenile paratypes.

Voucher numbers: DECBA1788, DECBA1789, DECBA1790, DECBA1796.

###### Collection/ecological information.

All additional juvenile individuals reported here were collected in leaf litter pitfall traps baited with beer in an uplands secondary forest at CEIBA biological station.

###### Summary of juvenile morphology.

Juveniles are apterous and largely match the morphology of adults except for in the following. Simple styli present on the subgenital plate in some individuals but are short and abbreviated. Spines on ventro-anterior margin of forelimb are as follows: 12 to 14 spines decreasing in size basally to apically, one or two slightly larger preapical spines and one large apical spine making a sum total of 15 or 16 total spines.

###### Molecular data and evolutionary placement.

Vouchers numbers and GenBank accession numbers: DECBA1791 – KF155114, DECBA1789 – KF155105, DECBA0801 – CBA0801, DECBA1827 – KF155103, DECBA1826 – KF155107, DECBA1814 – KF155115. The clade containing the above haplotypes (formerly reported as “Blattodea sp.1”) is supported by 96% bootstrap support and the haplotypes are nearly identical.

###### Diagnostic features of *Xestoblatta berenbaumae*.

The morphology of modified styles on the subgenital plate is the most useful trait for discerning this species with other *Xestoblatta* Hebard, 1916. The simple dorsal tergal gland, shape of the paraprocts (left modified into a tri-dentate or bi-dentate spine), and morphology of the internal genital sclerites of the male are also useful in identifying this species. Unfortunately the adult females and juveniles are largely lacking obvious identifying characteristics and there may be errors made in associating juveniles to the adults without the use of genetic information.

###### Etymology.

We give this species the specific epithet “*berenbaumae*” in honor of the esteemed entomologist, Dr. May Berenbaum, who has made huge contributions to entomology through scientific products, service and public outreach.

###### Known geographic distribution.

Guyana

**Table 3.** Allometry of *Xestoblatta berenbaumae* sp. n. All values are lengths reported in millimeters.

Morphological feature			*Xestoblatta berenbaumae* sp. n.										
			Adult ♂ (HT)	Adult ♂	Adult ♂	Adult ♂	Adult ♂	Adult ♂	Adult ♂	Adult ♀	Adult ♀	Adult ♀	Adult ♀
			DECBA2109	DECBA1967	DECBA0801	DECBA1958	DECBA2182	DECBA2092	DECBA2039	DECBA1787	DECBA1974	DECBA1793	DECBA2074
Head	Greatest width		2.7	3.0	3.0	2.9	3.0	2.8	2.9	3.1	3.0	3.0	3.1
	Medial length		3.6	3.8	3.8	3.8	3.9	3.5	3.6	4.0	4.0	4.0	3.7
Pronotum	Greatest width		5.3	6.0	6.0	5.6	4.9	5.3	5.3	5.9	4.7	5.9	4.1
	Medial length		4.0	4.0	4.4	4.0	4.5	4.0	4.0	4.8	5.9	4.3	5.6
Leg	Front	Femur	3.0	3.5	4.0	4.0	3.3	3.2	4.0	3.0	3.6	3.5	3.0
		Tibia	2.0	2.0	2.5	2.0	2.5	2.0	2.4	2.0	2.6	2.3	2.0
	Middle	Femur	4.0	4.5	4.2	4.0	4.7	4.0	4.8	4.2	4.3	4.4	4.2
		Tibia	3.8	3.7	4.0	4.0	3.7	4.0	4.4	4.0	4.0	3.8	4.0
	Hind	Femur	4.9	5.0	5.0	4.5	5.0	4.8	5.4	5.7	5.0	4.9	5.0
		Tibia	6.0	6.1	6.0	5.6	6.0	6.1	6.0	6.2	6.1	6.0	6.3
Cerci length			2.8	3.0	NA	2.3	2.5	3.2	3.0	3.0	2.8	2.4	NA
Tegminal length			13.5	14.0	14.0	14.0	13.3	14.0	13.8	10.1	10.0	10.0	9.2
Total body length			NA	NA	NA	17.5	15.4	16.0	18.0	18.7	17.0	18.2 (est.)	NA

##### Xestoblatta agautierae

Taxon classificationAnimaliaBlattodeaEctobiidae

Grandcolas, 1992

###### Materials.

*Adult* ♂.

Voucher number: DEKBO0827.

Collection locale. Wilson’s pond trail (Honey pond trail), Karanambu Ranch, Rupununi, Guyana.

GPS: 3°44'42.36"N, 59°19'15.21"W.

Collection date: 10 – June – 2013.

Collectors. Dominic A. Evangelista, Oswin Ambrose, Susan George, and Megan M. Wilson.

*Adult* ♀.

Voucher number: DEKBO0826.

Collection locale. Forest Island “Darwin”, Karanambu Ranch, Rupununi, Guyana.

GPS: 3°47'47.62"N, 59°22'6.77"W.

Collection date: 14 – June – 2013.

Collectors. Dominic A. Evangelista, Oswin Ambrose, Susan George, and Megan M. Wilson.

###### Collection/ecological information.

Both specimens above were collected in pitfall traps baited with beer in the forests of the Rupununi savannah.

###### Morphological identification.

**Figure 11.**
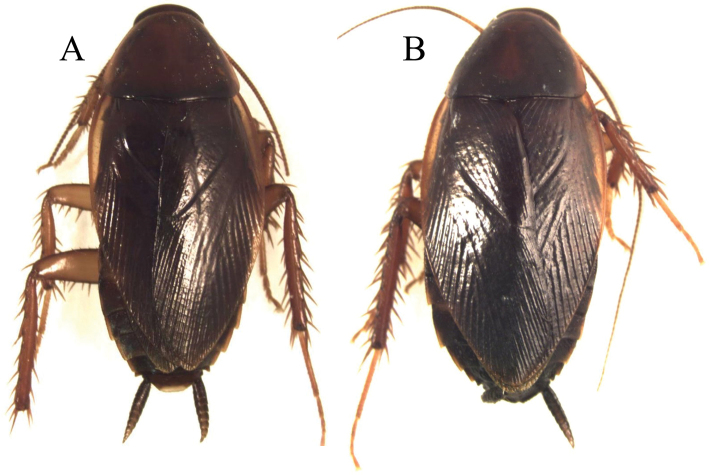
*Xestoblatta agautierae.* **A** Adult male dorsal view (DEKBO0442) **B** Adult female dorsal view (DEKBO0445).

The left genital phallomere, right genital phallomere, absence of a dorsal tergal gland and body coloration match closely with the species description (Grandcolas 1992a). The styli differ slightly to the illustrations in the original description in that the left stylus of our specimen is shorter and originates more medially. The female was associated to the male by comparison of gross morphology and body coloration. See Figure 11 for photos of adult male and adult female.

###### Collection/ecological information for other specimens not reported here.

We collected many individuals of this species from most forested areas surrounding Karanambu Ranch. We collected only one individual of this species in a similar trap at the edge of a forest, near open savannah. We found this species and *Xestoblatta berenbaumae* sp. n. to be extremely abundant in their respective localities (>100 individuals of each collected). However, both are previously unreported for Guyana. We believe this can be attributed to the fact that we used beer and fermenting fruit to bait out pitfall traps. As Gurney (1939) reports, *Xestoblatta* Hebard, 1916 were rare in collections until the contributions of an entomologist trapping fruit flies in Panama. We can speculate that these fruit flies were also collected with some sort of aromatic bait (as this is common for fruit fly trapping) that attracted the *Xestoblatta* Hebard, 1916 as by-catch.

###### Known geographic distribution.

Guyana (new record) and French Guiana.

#### Subfamily: Nyctiborinae

##### Nyctibora dichropoda

Taxon classificationAnimaliaBlattodeaEctobiidae

Hebard, 1926

###### Materials.

**Figure 12.**
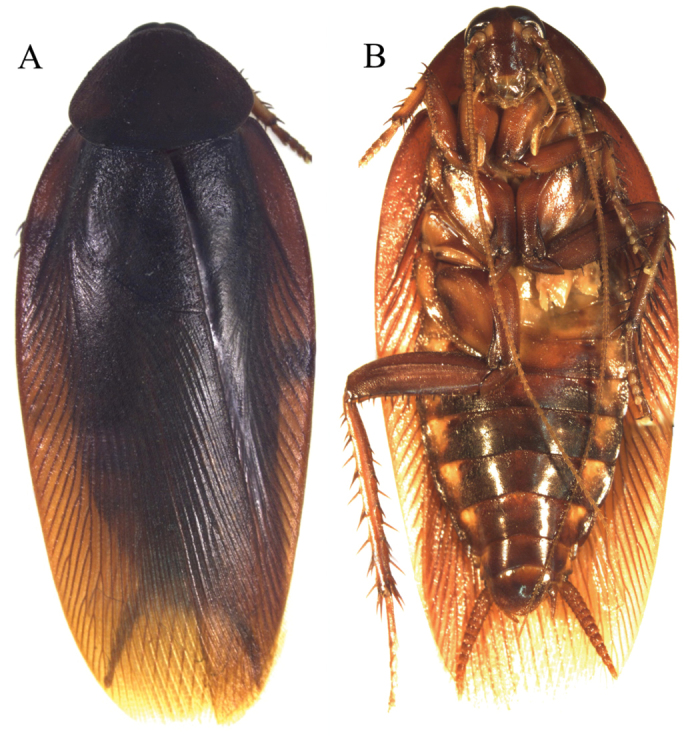
*Nyctibora dichropoda* adult male (DECBA0302). **A** Dorsal view **B** Ventral view.

*Adult* ♂ Figure 12.

Voucher number: DECBA0302.

GenBank accession number: KF155061.

Collection locale. CEIBA Biological Station, Madewini, Guyana.

GPS: 6°29'N, 58°13'W.

Collection date: 29 – July – 2011.

Collectors. Dominic A. Evangelista, Ian Biazzo, Manpreet K. Kohli, Melissa Sanchez-Herrera, Nicole Sroczinski and Jessica L. Ware.

###### Collection/ecological information.

This specimen was collected in the leaf litter.

###### Morphological identification.

This specimen matches the illustration and description by Hebard (1926) in the “striking pale” coloration on the surfaces of the tibiae, the definitive character for this species. However, the male we have is much larger than that which he described. It is matching in all other ways.

###### Molecular identification.

The COI barcodes of this specimen are close to an adult female (Voucher number: DECBA0235; GenBank accession number: KF155062) and juvenile specimen (Voucher number: DECBA0104; GenBank accession number: KF155024) of *Nyctibora*. Based on both genetic distance and morphological dissimilarity, these individuals are likely members of a separate species. We do not report them further here.

###### Known geographic distribution.

Guyana (new record), Suriname and French Guiana.

#### Subfamily: Pseudophyllodromiinae

##### Chorisoneura inversa

Taxon classificationAnimaliaBlattodeaChorisoneura

Hebard, 1926

###### Materials.

**Figure 13.**
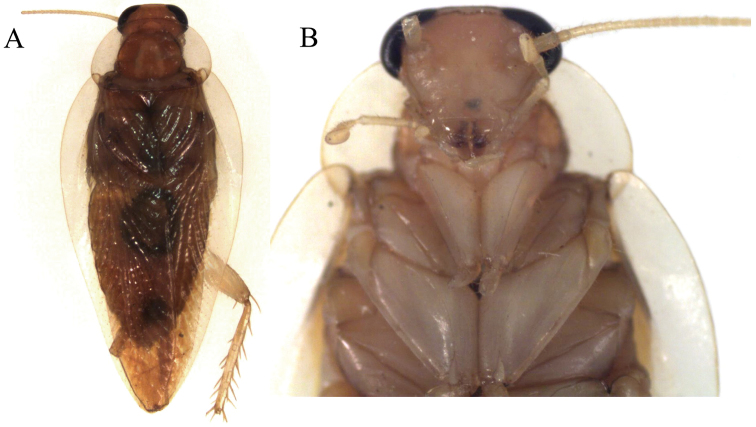
*Chorisoneura inversa* adult male (DECBA1782). **A** Dorsal view **B** Ventral view of head.

*Adult* ♂ Figure 13.

Voucher number: DECBA1782.

GenBank accession number: KF155130.

Collection locale. CEIBA Biological Station, Madewini, Guyana.

GPS: 6°29'N, 58°13'W.

Date: 7 to 11 – August – 2013.

Collectors. Dominic A. Evangelista, Ian Biazzo, Manpreet K. Kohli, Melissa Sanchez-Herrera, Nicole Sroczinski and, Jessica L. Ware.

###### Morphological identification.

This individual was recognizable when comparing to the description of Hebard (1926) and the presence of the anteriorly pointing “V” shape on pronotum.

###### Genetic information and evolutionary placement.

As discussed below, this specimen was placed near *Calhypnorna* Saussure & Zehntner, 1893 with 75% bootstrap support.

###### Known geographic distribution.

Guyana, Suriname, French Guiana and Brazil.

##### Dendroblatta callizona

Taxon classificationAnimaliaBlattodeaEctobiidae

Rehn, 1928

###### Materials.

**Figure 14.**
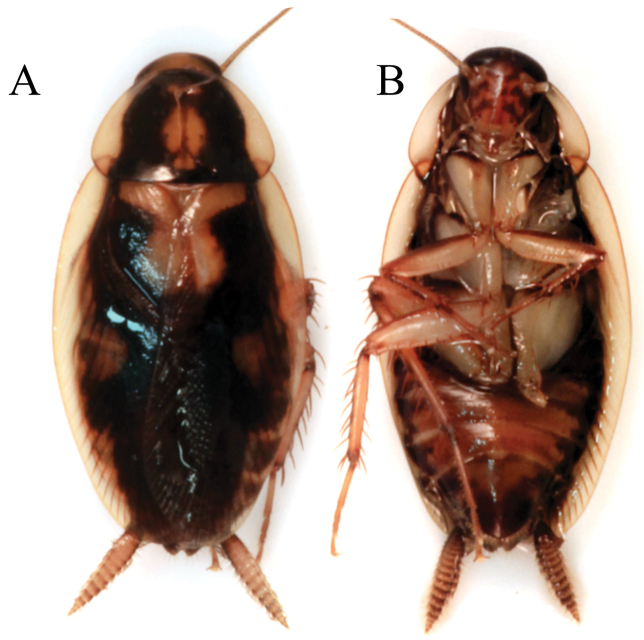
*Dendroblatta callizona* adult female (DECBA0805). **A** Dorsal view **B** Ventral view.

*Adult* ♀ Figure 14.

Voucher number: DECBA0805.

Collection locale. CEIBA Biological Station, Madewini, Guyana.

GPS: 6°29'57.75"N, 58°13'7.28"W.

Date: 14 – August – 2011.

Collectors. Dominic A. Evangelista, Ian Biazzo, Manpreet K. Kohli, Melissa Sanchez-Herrera, Nicole Sroczinski, and Jessica L. Ware.

Juvenile

Voucher number. DECBA0901.

GenBank accession number: KF155067.

Collection locale. CEIBA Biological Station, Madewini, Guyana.

GPS: 6°29'57.75"N, 58°13'7.28"W.

Date: 13 – August – 2011.

Collectors. Dominic A. Evangelista, Ian Biazzo, Manpreet K. Kohli, Melissa Sanchez-Herrera, Nicole Sroczinski, and Jessica L. Ware.

###### Collection/ecological information.

Both of these specimens were collected in a cup baited with beer placed in the canopy. The cup was tied to the trunk of a tree 13.8 meters above the ground. The tree chosen was close to a swampy primary forest area and on the edge of grassy hillside (most likely a plot that had been burned in the past). There were traps placed in the same tree at other heights but both individuals of this species were caught in this particular trap.

###### Morphological identification.

Our female specimen of *Dendroblatta callizona* Rehn, 1928 is within the variation described by Rehn (1928). The juvenile specimen was identified by comparison with the adult and using genetic data as well.

###### Genetic information.

In the tree of Evangelista et al. (2014) this species is placed near two individuals reported as “Ectobiidae sp. 10”. The morphology of these specimens is consistent with *Dendroblatta cnephaia* Hebard, 1926, although we do not report them here because of a lack of adults to confirm identification.

###### Known geographic distribution.

Trinidad and Tobago, Guyana, and Suriname.

##### Calhypnorna sp. A

Taxon classificationAnimaliaBlattodeaEctobiidae

###### Authors of the description.

Evangelista, Wilson, & Ware.

###### Materials.

**Figure 15.**
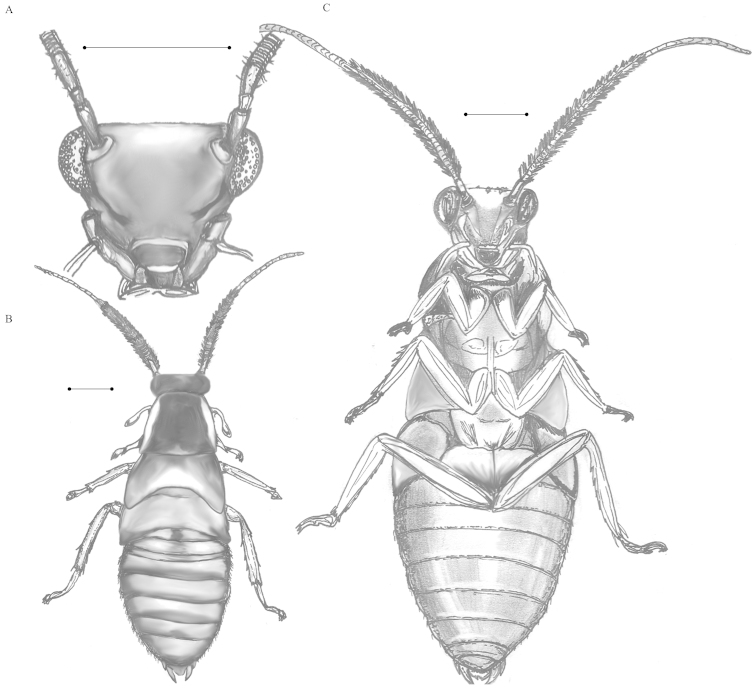
*Calhypnorna* sp. **A** Ventral view of head **B** Dorsal view of body **C** Ventral view of body. Scale bar = 1 mm. Illustrations contributed by Megan M. Wilson.

*Juvenile* Figure 15.

Voucher number: DECBA1802.

GenBank accession number: KF155118.

Collection locale. CEIBA Biological Station, Madewini, Guyana.

GPS: 6°29'N, 58°13'W.

Collection date: 15 – August – 2012.

Collectors. Dominic A. Evangelista and William R. Kuhn.

###### Specimen information.

This specimen is stored in ethanol and is deposited in the Center for Biodiversity at the University of Guyana.

###### Identification and differential diagnosis.

We identified this specimen as *Calhypnorna* based on the following comparisons. Our specimen is not lacking an interocular carina as in *Hypnornoides* (Rehn 1917). Our specimen also has a definitively truncate posterior margin of the pronotum (Figure 15B), which differentiates it from *Euhypnorna* (Hebard 1921). Our specimen is lacking the hairs covering most of the body as in *Hypnorna* (1893) and most closely matches the illustration of *Calhypnorna* by Saussure & Zehntner (1893).

###### Description.

The specimen is a juvenile that is likely in its penultimate instar. Overall, the body shape is elongated for a typical cockroach, and even for a typical Pseudophyllodromiinae. A large portion of the head is visible from a dorsal perspective, and reaches anteriorly past the pronotum significantly. The black coloration on the pronotum is the same width as the width of the head where it meets with the pronotal margin (Figure 15B).

Antennae are hirsute to nearly plumose. The antennae are slightly clubbed basally with the widest point occurring at first segment of the flagellum. There are two major color regions of the antennae: a dark basal region and a light distal region. The dark basal region begins as slightly lighter than the remainder but becomes a dark black color by the end of the dark region. The 25^th^ segment of the antennae is the final dark segment. The 26^th^ antennal segment begins the light region of the antennae. The 26^th^ or 27^th^ and subsequent segments are nearly white, becoming more brownish orange after the 7^th^ white segment (33 total). The total number of antennal segments on the specimen is 38 (left) and 44 (right).

The head is very large in relation to the remainder of the body, triangular, and wider than typical for a Pseudophyllodromiinae (Figure 15A). Inter-ocular space is sharply angled creating a carina that begins where the compound eye meets the antennae. The antennal pits are closer together than the eyes. Eyes are prominent and appear to bulge the head laterally. Facial grooves spanning from the posterior portion of the eye towards the mouthparts are prominent. Coloration on head is brown-orange overall with a slightly lighter, less brown, patch above and below the carina. Ocellar spots are either absent or not readily visible.

The pronotum is colored with a dark black region taking up the major two fifths of the medial area. The black area is opaque and reaches forward to the anterior margin but just stops short of completion in the posterior eighth of the segment. The black region is nearly rectangular, slightly rounded anteriorly and widened posteriorly (Figure 15B). Bordering the black region laterally and posteriorly are translucent regions colored brown-orange similar to the remainder of the body.

Meta- and meso-thoracic segments are both strongly lobed, presumably due to the developing wings within. Color is orange-brown overall with small amounts of black on the tips of the posterior pair of wing pads. Legs are light in color with a slight orange tinge overall. Dark regions are present on the medial side of the base of the fore-coxae.

The ventro-anterior margin of the fore-femur have five (right) or eight (left) large piliform spines basally followed by 27 (right) and 20 (left) shorter piliform spines, which are then each followed by one larger piliform spine and finally one large distal spine that is not piliform. Arolia are large and extend beyond the tips of the pretarsal claws on all legs. Claws are symmetrical and unspecialized.

Both the venter and dorsum of the abdomen is the same orange-brown color as the remainder of the body, but with a slightly redder tinge. Soft black color borders the abdomen laterally and posteriorly.

The dorsal abdomen is mostly glabrous. Hairs that are present are most dense laterally and on segments five and six. Ventral abdomen is glabrous as well, with fewer hairs than on the dorsal side and no regions with any dense pubescence. Supra-anal plate is unspecialized and broadly subtrapezoidal or triangular. Subgenital plate is broadly subtrapezoidal with the posterior margin being broader than that of the subgenital plate. The posterior margin of the subgenital plate is not perfectly uniform and conforms around two large styli. Styli are equal in length to the entire subgenital plate. Their width is equal to half of the length of the visible portion of the styli.

###### Genetic information and evolutionary placement.

Evangelista et al. (2014) recovers this sequence as being most closely related to a species reported as “Ectobiidae sp. 6” with 75% bootstrap support. This species is identified above as *Chorisonuera inversa* Hebard, 1926. Hebard hypothesized that these are closely related genera (Hebard 1921a) and we can now say that genetic data supports this hypothesis. We cannot definitively say, however, that they are sister taxa because of incomplete phylogenetic sampling in this tree. Thus, we follow Hebard (1921a) and not Beccaloni (Beccaloni 2007) and consider this to be in the Psuedophylodromiinae.

###### Known geographical distribution of Calhypnorna.

Guyana (new record), Para Brazil, Bolivia and Panama.

###### Collection/ecological information.

This specimen was found crawling through a benab. The only individual of this species observed in the field was the one collected and described here. Given that our overall collecting effort was significant (>1000 individuals of Blattodea *s.s.*) and we only found a single individual of *Calhypnorna* sp. A, we consider this species to be quite rare.

**Figure 16.**
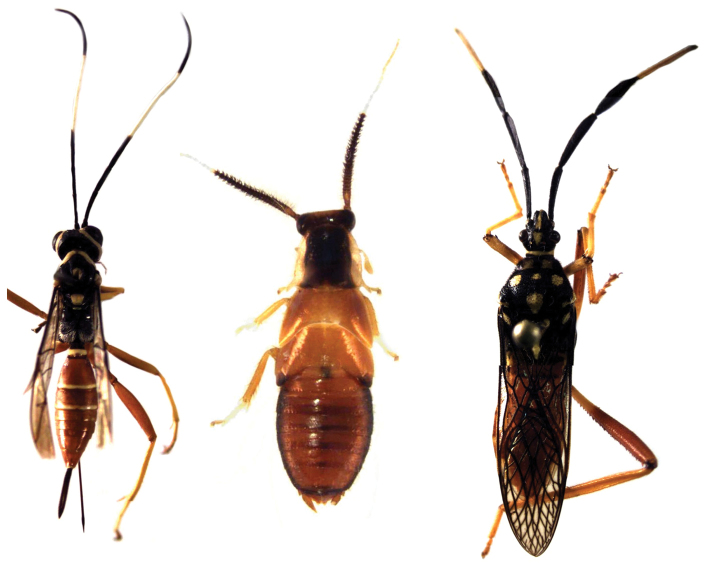
Comparison of overall body coloration of three sympatric species (Left: Ichneumonidae, Middle: *Calhypnorna* sp. “Aguyana”, Right: Reduviidae) from northern Guyana. Calhypnorna sp. shares the orange hind section and dark forward section with the other two insects. Additionally, the antennae of the cockroach composed of: a white band shared with the wasp; an orange band shared with the assassin bug; and a black base share among all. Photos are not to scale.

Previous work (Shelford 1912) has cited species of this genus as being beetle mimics. However, we observed no beetle model in the field that this species may have been mimicking. We did notice a similarity in body coloration of a wasp and Hemipteran sympatric with this conspicuously colored Blattodea (Figure 16).

###### Notes on historical records of this genus.

The genus *Calhypnorna* Saussure & Zehntner, 1893 was originally established as a subgenus of *Hypnorna* Stål, 1860. It was then given generic status by Kirby (1904). The genera *Calhypnorna*, *Hypnorna*, *Hypnornoides* Rehn, 1917 and *Euhypnorna* Hebard, 1921 are thought to be closely related (Hebard 1921). These are known from a number of regions (Para and Rio de Janiero Brazil, Bolivia and Panama) but there are no records from the Guiana Shield. Therefore, a new record of this species from the coastal rainforests of Guyana is geographically disjointed from all other records of these taxa. On this basis alone, we might distinguish this specimen as a new species. However, since our lone specimen is a juvenile, we have limited morphological basis for differentiating this from known taxa. We refrain from establishing this as new species until adult specimens can be found but we still give a synopsis of the biological traits of this specimen. This new record extends the potential range of *Calhypnorna* Saussure & Zehntner, 1893 and it has now been recorded from Para Brazil (south of Amazon), Bolivia, Panama, and Guyana (new record).

### Cockroach fauna of the Guyana Shield: Summary

**Figure 17.**
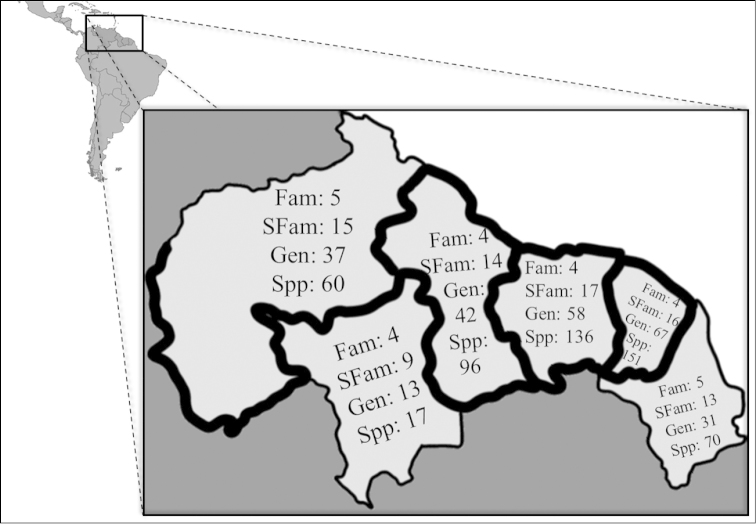
Known richness of cockroach fauna at different taxonomic levels for six regions of the Guiana Shield.

The checklist (Table 1) contains 5 families, 18 subfamilies, 79 genera, and 234 species. French Guiana and Suriname contribute the most to this richness, with 151 and 136 species respectively (Figure 17). The surprisingly low number of records from Guianan Venezuela, Roraima and Amapa Brazil (Figure 17) are most definitely due to an historical under sampling in these regions.

**Figure 18.**
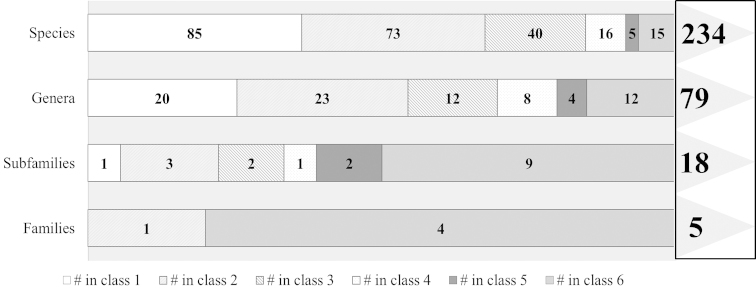
Extent of range for cockroach taxa. Classes represent the number of regions a taxon was present in: present in only one region – class one; present in all six regions – class 6; etc. Total number of taxa for each level shown on the right.

When pooling and examining the range data for all the taxa (Figure 18) we see that, as expected, small ranges are most common among species. This is also true when pooling taxa together into genera, although these range sizes are larger overall. 85 species (36%) and 20 genera (25%) are limited to a single region while 36 species (15%) and 24 genera (30%) are represented in four or more regions. Small ranges (<4 regions) are no longer the majority when lumping species into subfamilies or families.

**Figure 19.**
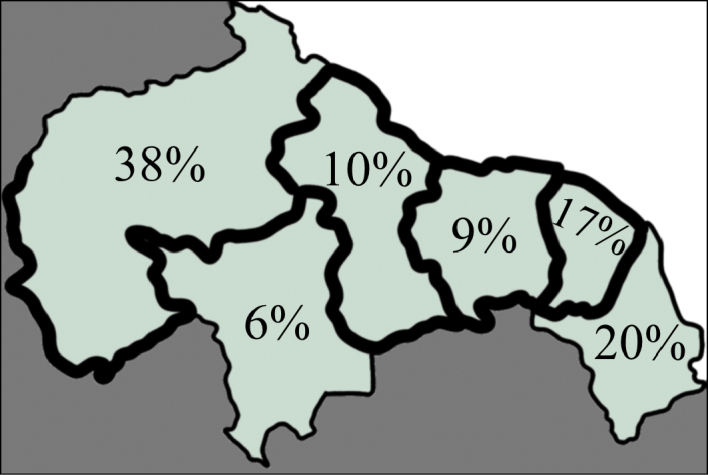
Proportion of cockroach fauna endemic to a region. Endemism is only referred to within the context of the shield.

**Figure 20.**
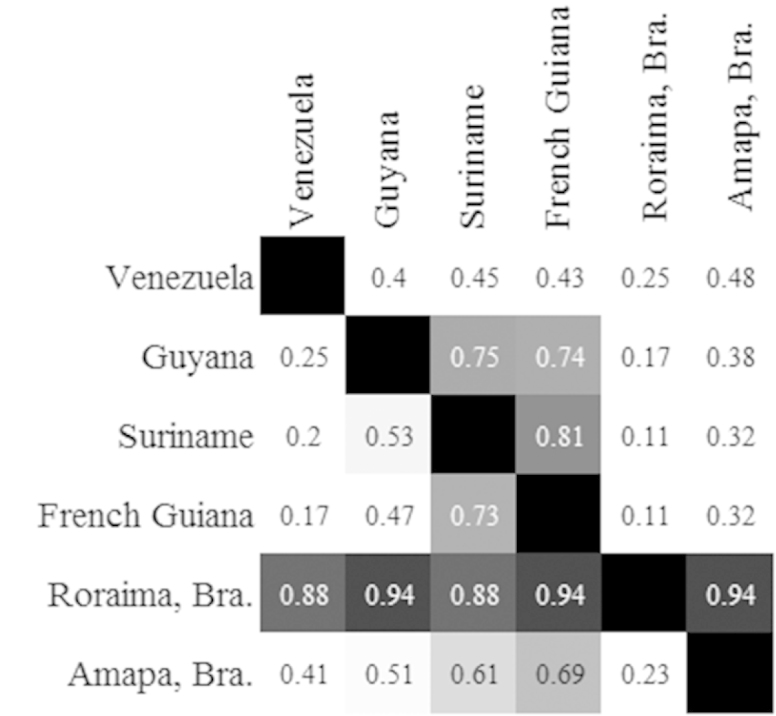
Proportion of fauna in a region (left) shared with each other region (top). Values greater than. 5 are shaded by magnitude. The three central regions (Guyana, Suriname and French Guiana) have a high degree of similarity with each other.

The highest rates of endemism are seen in Guianan Venezuela, Amapa Brazil and French Guiana (Figure 19). However, we believe these values to be inaccurate due to lack of sampling. Compared on a pairwise basis, Guyana, Suriname and French Guiana had a high proportion of shared fauna (Figure 20). These are each proximal to each other and centrally located, thus their faunal similarity is expected. Roraima showed a high number of its own species shared among each other region. However, most of the species recorded from Roraima are circumtropical taxa and the region is severely under sampled.

Most of the species in the checklist have neotropical distributions. There were few taxa listed with distributions that may be considered circumtropical or cosmopolitan: *Blatta orientalis* Linnaeus, *Neostylopygia rhombifolia* (Stoll), *Periplaneta americana* (Linnaeus), *Periplaneta australasiae* (Fabricius), *Periplaneta brunnea* Burmeister, *Holocompsa nitidula* (Fabricius), *Phoetalia pallida* (Brunner von Wattenwyl), *Phoetalia circumvagans* (Burmeister), *Nauphoeta cinerea* (Olivier), *Rhyparobia maderae* (Fabricius), *Panchlora nivea* (Linnaeus), *Pycnoscelus surinamensis* (Linnaeus), *Blattella germanica* (Linnaeus), *Supella longipalpa* (Fabricius). Most of these may be considered non-native, or adventive.

**Figure 21.**
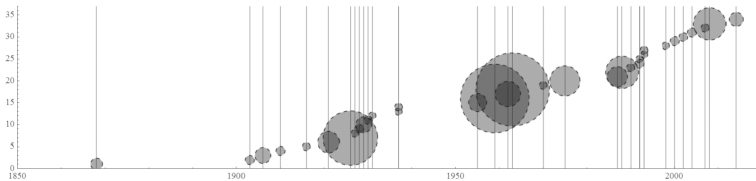
Studies contributing to the checklist of cockroaches of the Guiana Shield. The year of publication of each source plotted against the order in which they were published. The present study, the 34th, is the final circle in the top right. The radius of the circles represents the relative number of times that study is cited in the checklist.

## Discussion

The majority of records used to compile the checklist were lacking in specific biological, geographic or ecological information. Most historical records we encountered only gave general collection locales within their respective country. GPS information was non-existent for nearly all records.

We present eight new species records for Guyana. This includes one genus new to the entire shield (*Calhypnorna* Saussure & Zehntner, 1893) and one new species (*Xestoblatta berenbaumae*). Given the somewhat high local richness of cockroaches (Evangelista et al. 2014) in one small plot compared to the richness of the entire country (Figure 17) we believe that much of this country’s diversity has yet to be discovered.

Among the regions considered here, Guyana and Amapa are moderately well sampled. Guianan Venezuela, and Roraima Brazil are sampled especially poorly and our knowledge of the Blattodea of these regions is very much preliminary. In contrast, French Guiana and Suriname are some of the most well sampled cockroach faunas in all the neotropics, ranking as the 2^nd^ and 6^th^ most species dense regions respectively (Table 4). The most well sampled region in the neotropics, Rio de Janeiro, has a species density of 0.01 species per square mile (Table 4). If we consider this value as being typical of true species density, which is purely speculation, then no other neotropical region has been sampled thoroughly.

The levels of endemism we see (Figure 19) are surprisingly low compared with other known rates of endemism for the Guiana Shield (Funk et al. 2007; Hollowell and Reynolds 2005; Kelloff and Funk 2004; Naka 2011). One possible explanation would simply be that cockroaches have low rates of tropical endemism. However, this is contradicted by other cockroach faunas showing much higher rates of endemism (e.g. ~60% of all taxa in Hispaniola; Gutierrez and Perez-Gelabert 2000). The alternate explanation is that there is a collection bias for taxa with broad ranges. This could be true if geographic sampling is very sparse, which may be the case. The levels of endemism we report (Figure 19) are actually higher than what they are in reality, since we only considered strictly Guianan regions. There are likely a few species that appear endemic when only considering these regions but by expanding the geographic scope we would find that they are actually not Guianan endemics (e.g. also being present in Trinidad, Colombia or other parts of Brazil).

If we didn’t already know that under-sampling for cockroaches (Pellens and Grandcolas 2008; Roth 2003) and other insects (Erwin 1982; Stork 1993) was generally problematic, we could infer this based on a number of clues in our data. First, as mentioned previously, an estimate of total species richness of cockroaches for one small plot in northern Guyana nearly matches the recorded richness of the entire country (Evangelista et al. 2014). Furthermore, there are 20 cases of species with unusual distributions (Table 5), where it is absent from a region but recorded from neighboring regions. Without evidence to the contrary, the simplest explanation for these distribution “holes” is inadequate sampling. Finally, although specific locality information is severely lacking for most records, those that are recorded do not represent effective spatial sampling, and most records are from coastal areas of major rivers. Finally, the number of species per region is significantly lower than that of better sampled but less diverse taxa such as Odonata (“Checklist of Odonata of the Guiana Shield” 2012; Garrison et al. 2006, 2010).

Although there is clearly a great under-sampling of cockroaches from this region, we cite 34 publications that contributed to this checklist, including the present (Figure 21). The earliest source was from 1868 (Walker 1868). Most of the publications contributing to the checklist were published between 1900 and 1940. Morgan Hebard, Isolda Rocha e Silva Albuquerque, Ashley Gurney and James Rehn contributed the most through primary taxonomic publications and species descriptions (in particular see Hebard 1926; Rehn 1930; Rocha E Silva Albuquerque and Gurney 1962). Karlis Princis, J. Bonfils and Conrad F.A. Bruijning were also important in these capacities but more-so through their own published checklists. Jaime Perez and J. Bonfils were also great contributors to the fauna of Venezuela and French Guiana. Similarly, Roseli Pellens was an important contributor to the knowledge of the two Brazilian regions through her checklist. Philippe Grandcolas was also an instrumental author through this same checklist, as well as other primary taxonomic publications. The three most cited papers in the checklist are Princis’ “Orthopterum Catalogus” (148 citations), Bruijning’s “The Blattidae of Surinam” (138 citations), and Hebard’s “The Blattidae of French Guiana” (105 citations) (Figure 21). It is worth restating that, although they are invaluable authors, Princis’ and Bruijning’s contributions were mainly through synthesizing work done by others. The significance of Hebard’s contribution to the knowledge of the Guianan fauna through “The Blattidae of French Guiana”, in which he alone described 53 new species, cannot be understated.

**Table 4.** The ten regions of the Neotropics with the highest known cockroach richness per unit area.

Region	Size (mi^2^)	# of spp.	spp/mi^2^	Source
Rio de Janeiro, Brazil	16,871	169	0.0100	(Pellens and Grandcolas 2008)
French Guiana	32,253	151	0.0047	-
Panama	29,118	118	0.0041	(Beccaloni 2007)
Costa Rica	19,730	72	0.0036	(Beccaloni 2007)
Hispaniola	29,530	86	0.0029	(Perez-Gelabert 2008)
Continental Ecuador	46,444	114	0.0025	(Vidlicka 2013)
Suriname	63,039	136	0.0022	-
Cuba	42,426	85	0.0020	(Gutierrez 1995)
Amapa, Brazil	55,141	70	0.0013	-
Guyana	83,000	96	0.0012	-

**Table 5.** Recorded (o and +) and projected (p) presences of cockroaches from the Guiana Shield. VEN – Combined data from Amazonas, Bolivar and Delta Amacuro Venezuela; GUY – Guyana; SUR – Suriname; FG – French Guiana; Rora BRA – Roraima, Brazil. Amapa BRA – Amapa, Brazil. Projected occurrences are expectations of species presence based on confirmed presence in neighboring regions. Data used to determine this is taken from the checklist (Table 1) and other sources (see Table 1 for citations for these species).

	*VEN*	*GUY*	*SUR*	*FG*	*Rora BRA*	*Amapa BRA*
**Blaberidae**						
**Blaberinae**						
*Blaberus colosseus*	**p**	o	**p**	o		
*Blaberus craniifer*	**p**	o	**p**	o		
**Epilamprinae**						
*Epilampra azteca*	o	**p**	o	o		
*Epilampra maculicollis*		o	**p**	o		
**Panchlorinae**						
*Panchlora bidentula*	o	**p**	o	o		
**Zetoborinae**						
*Thanatophyllum akinetum*		+	**p**	o		
**Ectobiidae**						
**Anaplectinae**						
*Anaplecta subsignata*	o	**p**	o	o		o
*Maraca fossata*	o	**p**	o	o		
**Blattellinae**						
*Cahita misella*	**p**	**p**	**p**	o		
*Chromatonotus notatus*	**p**	**p**	o	o		
*Eudromiella ineopectata*		o	**p**	o		
*Xestoblatta nyctiboroides*		o	**p**	o		
*Xestoblatta agautierae*		+	**p**	o		
**Pseudophyllodromiinae**						
*Anisopygia decora*		o	**p**	o		
*Arawakina frontalis*		o	**p**	o		
*Chorisoneura gatunae*	**p**	**p**	o	o		
*Euphyllodromia chopardi*		o	**p**	o	o	o
*Neoblattella guianae*		o	**p**	o		o
*Sciablatta poecila*		o	**p**	o		
*Trioblattella callosoma*		o	**p**	o		o

## Conclusions

This checklist of Blattodea s.s. of the Guiana Shield, showing 234 species, is the most comprehensive to date. It is also functions as the first true checklist of cockroaches of Guyana, as all previous sources severely fall short of listing even the modest number of species we record here. Given the large number of species found in the small country of French Guiana, we see that the Guiana Shield may be one of world’s hotspots of biodiversity for cockroaches. However, sampling is still severely lacking. What little sampling has been done in the Guianas was mostly completed before 1960. There are huge gaps to fill in, and until they are we will be unable to adequately address most questions about the nature and origins of cockroach biodiversity.

## Supplementary Material

XML Treatment for Eublaberus distanti

XML Treatment for Neorhicnoda maronensis

XML Treatment for Colapteroblatta surinama

XML Treatment for Epilampra opaca

XML Treatment for Epilampra sodalis

XML Treatment for Thanatophyllum akinetum

XML Treatment for Anaplecta parviceps

XML Treatment for Anisopygia decora

XML Treatment for Ischnoptera atrata

XML Treatment for Xestoblatta berenbaumae

XML Treatment for Xestoblatta agautierae

XML Treatment for Nyctibora dichropoda

XML Treatment for Chorisoneura inversa

XML Treatment for Dendroblatta callizona

XML Treatment for Calhypnorna sp. A
